# Geospatial Resolution of Human and Bacterial Diversity with City-Scale Metagenomics

**DOI:** 10.1016/j.cels.2015.01.001

**Published:** 2015-03-03

**Authors:** Ebrahim Afshinnekoo, Cem Meydan, Shanin Chowdhury, Dyala Jaroudi, Collin Boyer, Nick Bernstein, Julia M. Maritz, Darryl Reeves, Jorge Gandara, Sagar Chhangawala, Sofia Ahsanuddin, Amber Simmons, Timothy Nessel, Bharathi Sundaresh, Elizabeth Pereira, Ellen Jorgensen, Sergios-Orestis Kolokotronis, Nell Kirchberger, Isaac Garcia, David Gandara, Sean Dhanraj, Tanzina Nawrin, Yogesh Saletore, Noah Alexander, Priyanka Vijay, Elizabeth M. Hénaff, Paul Zumbo, Michael Walsh, Gregory D. O’Mullan, Scott Tighe, Joel T. Dudley, Anya Dunaif, Sean Ennis, Eoghan O’Halloran, Tiago R. Magalhaes, Braden Boone, Angela L. Jones, Theodore R. Muth, Katie Schneider Paolantonio, Elizabeth Alter, Eric E. Schadt, Jeanne Garbarino, Robert J. Prill, Jane M. Carlton, Shawn Levy, Christopher E. Mason

**Affiliations:** 1 Department of Physiology and Biophysics, Weill Cornell Medical College, New York, NY 10065, USA; 2 The HRH Prince Alwaleed Bin Talal Bin Abdulaziz Alsaud Institute for Computational Biomedicine, Weill Cornell Medical College, New York, NY 10065, USA; 3 School of Earth and Environmental Sciences, City University of New York (CUNY) Queens College, Flushing, NY 11367, USA; 4 CUNY Hunter College, New York, NY 10065, USA; 5 Center for Genomics, New York University, New York, NY 10003, USA; 6 Tri-Institutional Program on Computational Biology and Medicine (CBM), New York, NY 10065, USA; 7 CUNY Brooklyn College, Department of Biology, Brooklyn, NY 11210, USA; 8 Cornell University, Ithaca, NY 14850, USA; 9 Genspace Community Laboratory, Brooklyn, NY 11238, USA; 10 Department of Biological Sciences, Fordham University, Bronx, NY 10458, USA; 11 State University of New York, Downstate, Brooklyn, NY 11203, USA; 12 University of Vermont, Burlington, VT 05405, USA; 13 Icahn School of Medicine at Mount Sinai, New York, NY 10029, USA; 14 Rockefeller University, New York, NY 10065, USA; 15 Academic Centre on Rare Diseases, School of Medicine and Medical Science, University College Dublin 4, Ireland; 16 National Centre for Medical Genetics, Our Lady’s Children’s Hospital, Dublin 12, Ireland; 17 HudsonAlpha Institute for Biotechnology, Huntsville, AL 35806, USA; 18 CUNY York College, Jamaica, NY 11451, USA; 19 Accelerated Discovery Lab, IBM Almaden Research Center, San Jose, CA 95120, USA; 20 The Feil Family Brain and Mind Research Institute, New York, NY 10065, USA

## Abstract

The panoply of microorganisms and other species present in our environment influence human health and disease, especially in cities, but have not been profiled with metagenomics at a city-wide scale. We sequenced DNA from surfaces across the entire New York City (NYC) subway system, the Gowanus Canal, and public parks. Nearly half of the DNA (48%) does not match any known organism; identified organisms spanned 1,688 bacterial, viral, archaeal, and eukaryotic taxa, which were enriched for harmless genera associated with skin (e.g., *Acinetobacter*). Predicted ancestry of human DNA left on subway surfaces can recapitulate U.S. Census demographic data, and bacterial signatures can reveal a station’s history, such as marine-associated bacteria in a hurricane-flooded station. Some evidence of pathogens was found (*Bacillus anthracis*), but a lack of reported cases in NYC suggests that the pathogens represent a normal, urban microbiome. This baseline metagenomic map of NYC could help long-term disease surveillance, bioterrorism threat mitigation, and health management in the built environment of cities.

## Graphical Abstract

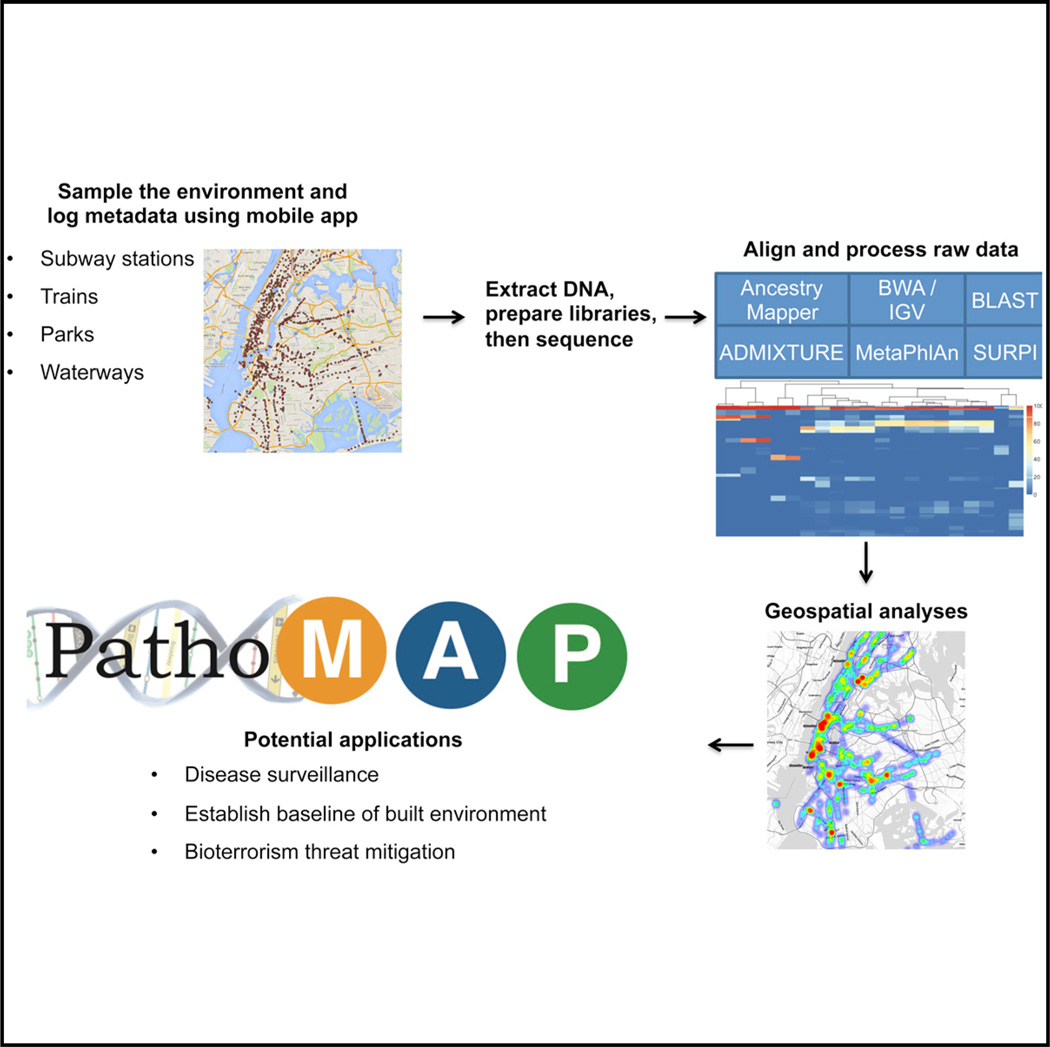

## INTRODUCTION

The microbiome represents the diversity of the microorganisms present in an environment, and the human microbiome has been increasingly recognized as an integral component of human health and disease (Peterson et al., 2009). In the average human, bacterial cells outnumber human cells by a 10:1 ratio (Qin et al., 2010), contribute as much as 36% of the active molecules present in the human bloodstream (Hood, 2012), and serve as a source of both pathogen protection (Vaarala, 2012) and risk (Markle et al., 2013). Thus, it is paramount to understand bacterial, viral, and metagenomic sources and distributions and how humans may interact with (or acquire) new commensal species or dangerous pathogens (Gire et al., 2014). This is especially important in dense human environments such as cities, wherein the majority of the world’s population (54%) currently live (The United Nations, 2014). Although environmental sequencing of targeted metropolitan areas that focused on the air (Robertson et al., 2013; Cao et al., 2014; Yooseph et al., 2013; Leung et al., 2014; Dybwad et al., 2014) or rodents (Firth et al., 2014) have been published, to our knowledge, the metagenomic geographic distribution of taxa from highly trafficked surfaces at a city-wide scale has not been reported.

**Figure 1:**
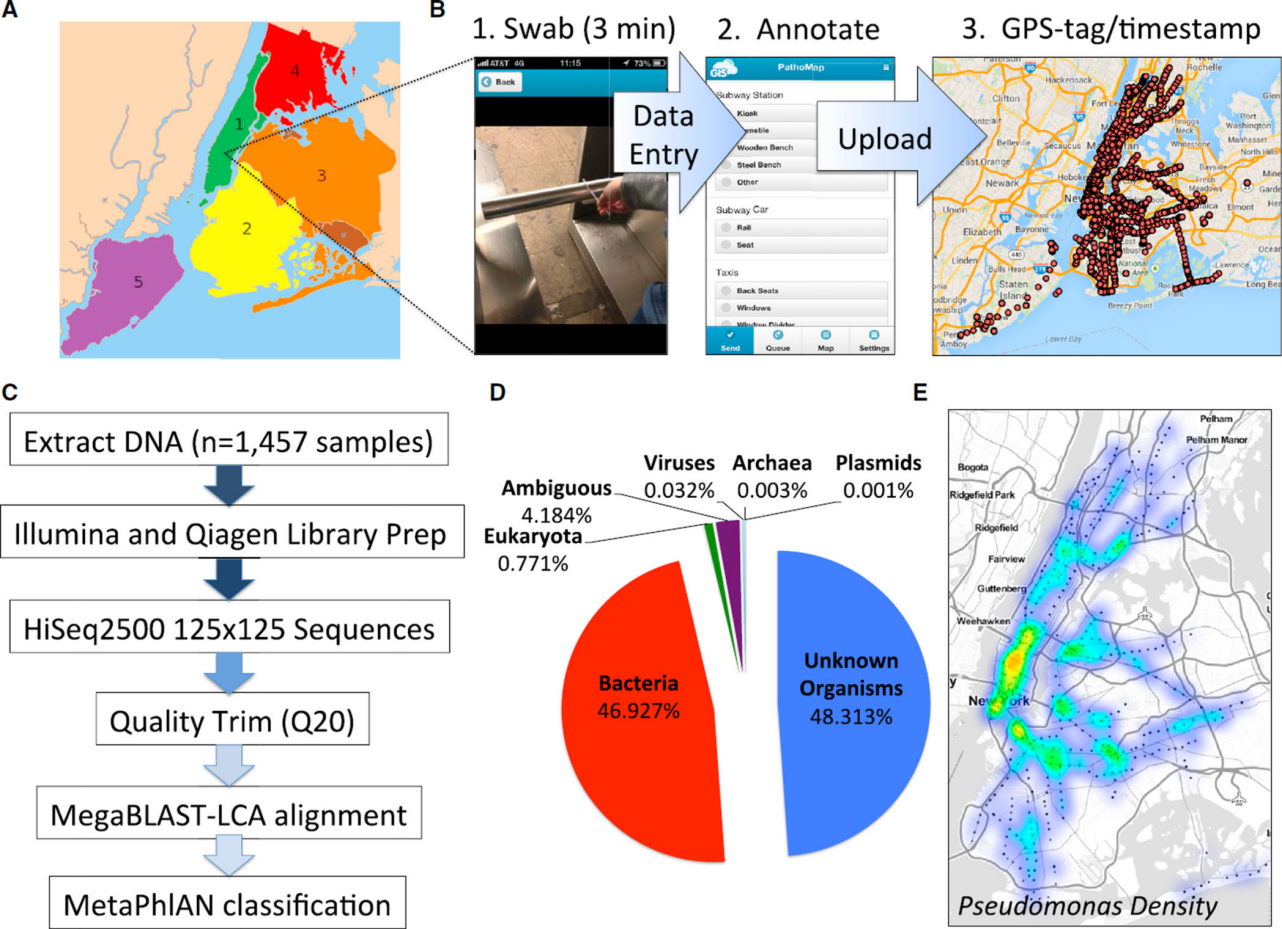
The Metagenome of New York City (A) The five boroughs of NYC include (1) Manhattan (green), (2) Brooklyn (yellow), (3) Queens (orange), (4) Bronx (red), (5) Staten Island (lavender). (B) The collection from the 466 subway stations of NYC across the 24 subway lines involved three main steps: (1) collection with Copan Elution swabs, (2) data entry into the database, and (3) uploading of the data. An image is shown of the current collection database, taken from http://pathomap.giscloud.com. (C) Workflow for sample DNA extraction, library preparation, sequencing, quality trimming of the FASTQ files, and alignment with MegaBLAST and MetaPhlAn to discern taxa present. (D) Distribution of taxa identified from the entire pooled dataset. (E) Geospatial analysis of the most prevalent genus, *Pseudomonas*, across the subway system; hotspots reveal high density of *Pseudomonas* in areas in Manhattan and Brooklyn.

The metropolitan area of New York City (NYC) is an ideal place to undertake a large-scale metagenomic study because it is the largest and most dense city in the United States; 8.2 million people live on a landmass of only 469 square miles (Figure 1A). Moreover, the subway of NYC is the largest mass-transit system in the world (by station count), spreading over 252 miles and used by 1.7 billion people per year (APTA Ridership Report, 2014). This vast urban ecosystem is a precious resource that requires monitoring to sustain and secure it against acts of bioterrorism, environmental disruptions, or disease outbreaks. Thus we sought to characterize the NYC metagenome by surveying the genetic material of the microorganisms and other DNA present in, around, and below NYC, with a focus on the highly trafficked subways and public areas. We envision this as a first step toward identifying potential bio-threats, protecting the health of New Yorkers, and providing a new layer of baseline molecular data that can be used by the city to create a “smart city,” i.e., one that uses high-dimensional data to improve city planning, management of the mass-transit built environment, and human health.

To describe, characterize, and track the microbiome and metagenome of NYC, we used next-generation DNA sequencing (NGS) technologies to profile the organisms present in our samples. We demonstrate the potential of these data for surveying the distribution of human alleles in a city and their intersection with orthogonal data like U.S. Census data. We also report here the validation and functional characterization of the samples collected, including ribosomal rRNA gene sequencing to complement the shotgun sequencing, culturing of the bacteria to test for the source of antibiotic resistance, and a characterization of some bacterial plasmids found in the bacteria. These data establish a city-scale, baseline metagenomic DNA profile, which is essential for subsequent work in contextualizing the potentially harmful, as well as neutral, bacteria and organisms that surround and move with human populations.

## RESULTS

### City-Scale Metagenomic Profiling

To create a city-wide metagenomic profile, we first built a mobile application (“app” for iOS and Android) in collaboration with GIS Cloud to enable real-time entry and loading of sample metadata directly into a database (Figure 1B). Each sample was geo-tagged with longitude and latitude coordinates via global positioning system (GPS), time-stamped, and photo-documented, and collection fields were completed for data entry and included the swabbing time, the scientist performing the collection, and collection notes (Figure 1B). This protocol enabled a built-in sample confirmation, where in we could confirm that the sample ID of the swab in the laboratory matched the ID in the photo taken during the collection.

We collected 1,457 samples across NYC. These included samples from all open subway stations (n = 466) for all 24 subway lines of the NYC Metropolitan Transit Authority (MTA), the Staten Island Railway (SIR), 12 sites in the Gowanus Canal, four public parks, and one closed subway station that was submerged during the 2012 Hurricane Sandy (Superstorm Sandy). At subway and railway stations, samples were collected in triplicate with one sample taken inside a train at the station and two samples from the station itself, with a serial rotation between the kiosks, benches, turnstiles, garbage cans, and railings (see Experimental Procedures). We obtained a median of 188 ng of DNA across all surfaces (Figure S1) in the city. We used shotgun sequencing to generate a total of 10.4 billion paired-end (125 3 125) DNA sequence reads, sequencing all samples to an average depth of 3.6M reads. Data were deposited and verified by the Sequence Read Archive (project PRJNA271013 and study SRP051511); all samples’ metadata and locations can be browsed at http://www.pathomap.org and in the (supplemental files.

We analyzed the metagenomic and microbial communities present in our samples using several tools (see detailed methods below). Briefly, all reads were first trimmed for 99% accuracy (Q value 20), followed by an alignment to all known organisms in NCBI with MegaBLAST-LCA (Wolfsberg and Madden, 2001) (lowest common ancestor [LCA] assignment by MEGAN) (Huson et al., 2007) and the Metagenomic Phylogenetic Analysis tool (MetaPhlAn v2.0) (Segata et al., 2012). Samples with predicted pathogens were further characterized with Sequence-based Ultra-Rapid Pathogen Identification (SURPI) (Naccache et al., 2014) and the Burrows-Wheeler Aligner (BWA) (Li and Durbin, 2010). A total of 21,885 and 1,688 taxa were assigned with MegaBLAST and MetaPhlAn, respectively, with 15,152 and 637 specific to the species level (Data Tables 1 and 2), respectively. Based on our sequencing of a positive control sample with titrated levels of known bacterial species (Figure S2; see Experimental Procedures), we set our thresholds of MegaBLAST and MetaPhlAn to enable an estimated minimum 99% specificity and 91% sensitivity for identifying taxa at the species level (Figure S3 and Tables S1 and S2).

We found that nearly half of the reads (48.3%) did not match to any known organism, underscoring the vast wealth of unknown species that are ubiquitous in urban areas (Figure 1D). These numbers are similar to the range recently reported for the “air microbiome” of NYC, where 25%–62% of sequenced DNA did not match any known organism (Yooseph et al., 2013). Of those reads assigned to an organism, we next separated out each species by abundance. The largest assigned category was for cellular organisms (48%), with most of these coming from bacteria (46.9% of all reads), followed by relatively small subsets of reads matching eukaryotes (0.8%), viruses (0.03%), archaea (0.003%), and plasmids (0.001%). The most prevalent bacterial species on the subway was *Pseudomonas stutzeri*, with enrichment in lower Manhattan (Figure 1E), followed by strains from *Enterobacter* and *Stenotrophomonas*. Notably, all of the most consistently abundant viruses were bacteriophages (Table 1), which were detected concomitant with their bacterial hosts in our dataset (Data Tables 1 and 2). These results demonstrate the ability of metagenomic data to help to confirm the presence of a bacterial species, as the phages provide a cross-kindgom mirror of the abundance of their hosts.

Human DNA was the fourth most abundant eukaryotic species, behind two insects, *Ceratitis capitata* (Mediterranean fruit fly) and *Dendroctonus ponderosae* (mountain pine beetle). Although these are the top-ranking matches according to a BLAST search for these reads (Table S3), the high incidence of *Dendroctonus ponderosae* may represent the presence of another, yet-to-be sequenced insect genome that is more prevalent in an urban, built environment (e.g., cockroaches are not yet in the NCBI data-base), given that these species share conserved genes like glycoside hydrolase (Eyun et al., 2014). Thus, although there is potential evidence for hundreds of other plants, fungi, and eukaryotic species in the subway (Data Table 1), the relatively few completed eukaryotic genomes focused our analysis on one of the best annotated genomes: the human genome.

### Human Allele Frequencies on Surfaces Mirror U.S. Census Data

**Figure 2:**
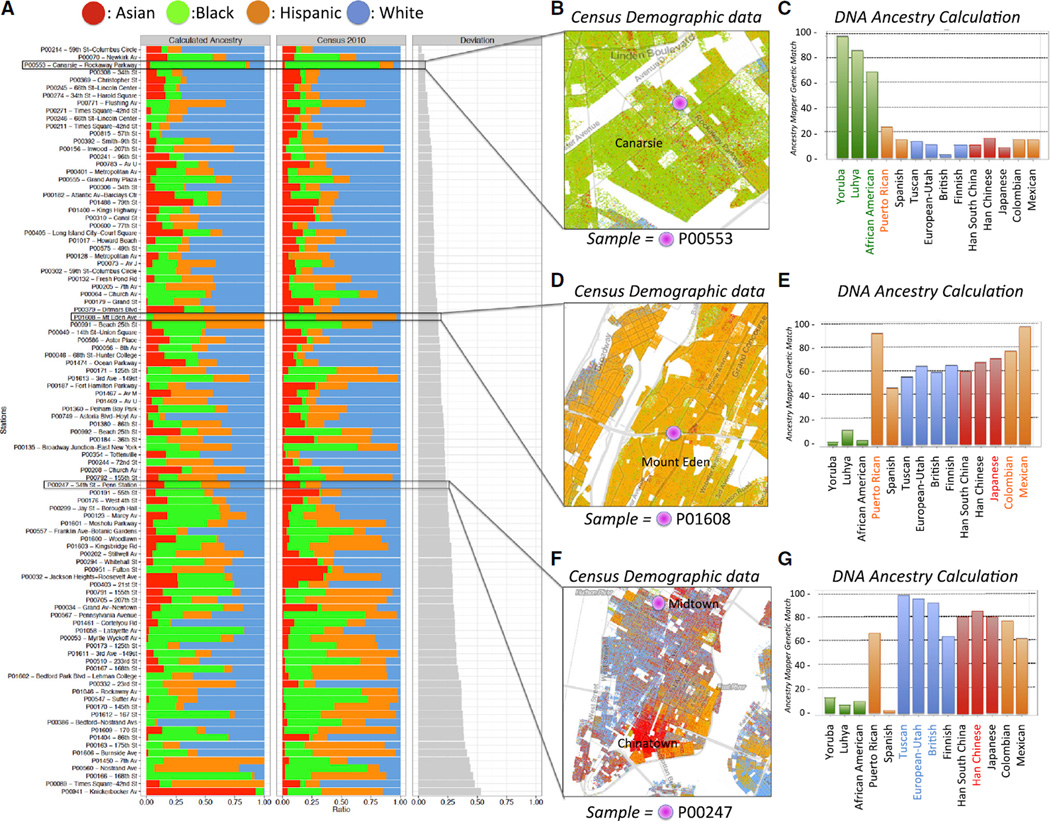
Human Ancestry Predictions from Subway Metagenomic Data Mirror Census Data Using ancestry-informative alleles from the 1000 Genomes Project and the ancestry prediction tool Ancestry Mapper, we were able to recapitulate the likely demographics of stations, based on the DNA left on the surfaces (A–G). We calculated the RMSD (gray bars) of the calculated ancestry versus the 2010 census data for each station (left). The colors for each ancestry are shown on top, and the stacked barplots show the proportion of 100% of alleles. We have used K=4 for admixture. In our datasets, the four ancestral components correspond to African/European/Asian/Ameridian. The Ameridian component has been matched to the Hispanic census designation; this is an approximation, as hispanics generally also have strong European components. For plots (B)–(G), horizontal black lines represent the percentage match (y axis) of alleles of each known an cestry (x axis); the top four ranking ancestries are highlighted using text labels colored to match census legends in (C), (E), and (G). In Canarsie, Brooklyn (B and C), an increase in African alleles was predicted, which matched the census data (green), and the same trend was observed for a primarily Hispanic area in the Bronx (Mount Eden). In one area of Manhattan near Penn Station, we found a higher incidence of European alleles concomitant with an increase in Asian alleles. Areas of the city (e.g., Chinatown) are annotated directly in the maps.

Despite sampling surfaces from areas of high human traffic and contact, we found that only an average of 0.2% of reads uniquely mapped to human genome with BWA (hg19, see Experimental Procedures). However, enough reads matched to the human genome to enable discovery of 5.3 million non-reference alleles from all samples across the city (Figure 2). We compared our sample collection map at pathomap. giscloud.com and with the predicted census demographics of the same GPS coordinate, using the 2010 U.S. Census Data (obtained from http://demographics.coopercenter.org). We hypothesized that the aggregate human genetic variants of a single subway station might echo the demographics of the reported population from the census data. We examined areas of NYC that showed a grouping in reported ethnicity (self-reported as White, Black, Asian, Hispanic) from all areas of an image-segmented U.S. Census Map (Figure S4) (Clinton et al., 2010), then compared these to samples wherein we observed enough human-mapping reads to call variants (see Supplemental Experimental Procedures). We then intersected these variants with ancestry-informative markers from the 1000 genomes (1KG) dataset, then used Ancestry Mapper (Magalhães et al., 2012) and Admixture (Alexander et al., 2009) to calculate the likely allelic admixture from the reference 1KG populations.

We observed that the human DNA from the surfaces of the subway could recapitulate the geospatial demographics of the city in U.S. Census data (Figures 2A–2G), relative to the reference populations used by Admixture and Ancestry Mapper. We found that the deviation from expected proportions of the calculated census data exhibited a wide range (Figure 2A), from nearly no deviation (root-mean-square deviation, RMSD = 0.03) to more discordant predicted/observed allele frequencies (RMSD = 0.53). For example, sample P00553 (Figure 2B) showed a majority African American and Yoruban ancestry for a mostly black area in Brooklyn (Canarsie), and this was nearly exactly calculated from the observed human alleles (Figure 2B). Also, in a primarily Hispanic/Amerindian area of the Bronx, Ancestry Mapper showed the top three ancestries to be Mexican, Colombian, and Puerto Rican (Figures 2D and 2E), which also correlated well with the human alleles. This site also showed an increase in Asian ancestry (Han Chinese and Japanese), which matches an adjacent area from the census data (Figure 2D). Finally, we observed that an area of Midtown Manhattan showed an increase in British, Tuscan, and European alleles, with some alleles predicted to be Chinese (Figure 2F), which also matches the census demographics of the neighborhood.

### Bacterial Genome Analysis Identifies Rare Potential Pathogens

We next investigated the bacterial content identified in our samples (Figure 1C), which generated a total of 1,688 bacterial taxa, with 637 of those specified down to the species level (Data Table 2). An annotation of the genus and species for our bacteria (Data Table 3) showed that the majority of the bacteria found on the surfaces of the subway (57%) are not associated with any human disease, whereas about 31% represent potentially opportunistic bacteria that might be relevant for immune-compromised, injured, or disease-susceptible populations. A smaller proportion (12%) of the detected taxa with species-level identification were known pathogens, including *Yersinia pestis* (Bubonic plague) and *Bacillus anthracis* (anthrax).

To further examine these putative pathogens, we focused only on species found by BLAST and MetaPhlAn and then compared our species to those annotated in the database of the National Select Agent Registry from the Centers for Disease Control (CDC) and the Pathosystems Resource Integration Center (PATRIC) lists of known pathogenic bacteria. At least three taxa on the CDC’s list of infectious agents and four organisms on the PATRIC list, including *Bacillus anthracis*, *Yersinia pestis*, and *Staphylococcus aureus*, showed evidence of being present in several stations, or dozens of stations (Table S4). It is worth noting that most strains of *E. coli* are benign, and these data do not (by themselves) indicate that these reads were from live pathogens. The presence of *E. coli*, however, indicates potential fecal contamination on surfaces or persons with the presence of *E. coli* skin infections, which is why it is listed on the PATRIC database.

**Figure 3:**
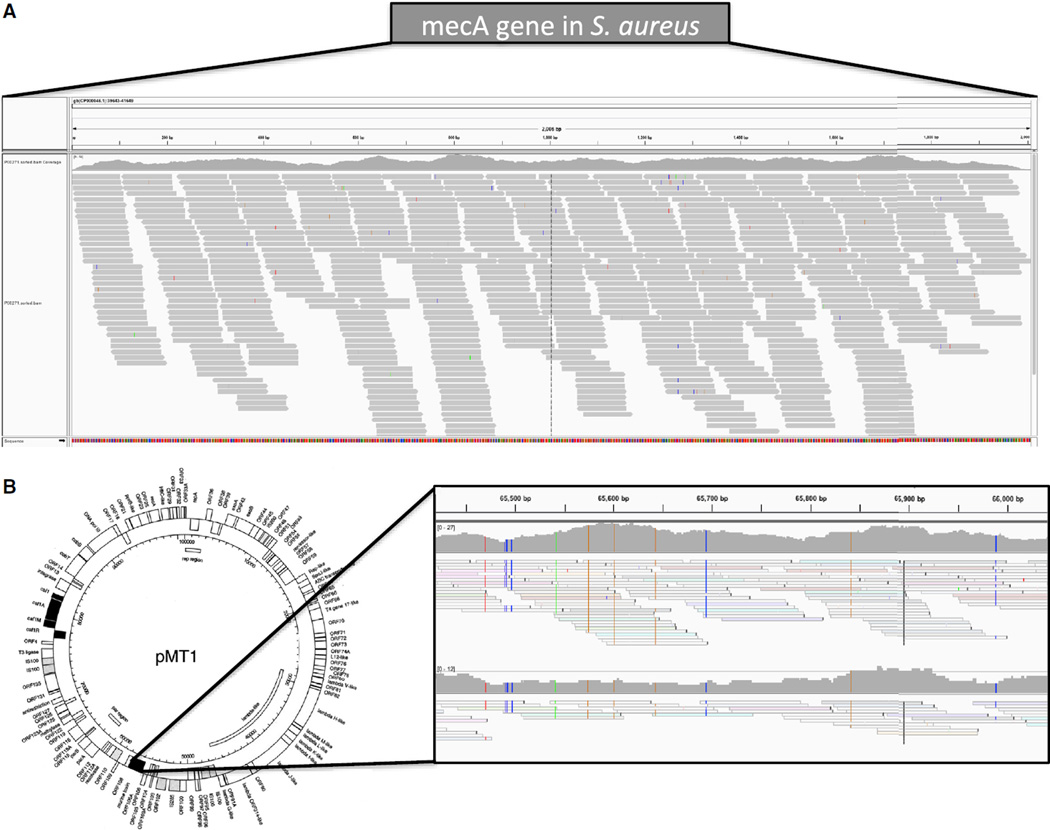
Coverage Plots of Virulence Elements from Staphylococcus aureus and Yesinia pestis We used the Integrative Genomics Viewer to plot the mapped number of reads from the shotgun sequence data that mapped to known virulence elements, including (A) the *mecA* gene from MRSA and (B) the pMT1 plasmid from *Y. pestis*. Coverage depth is shown at the top of each inset, with SNPs shown as vertical colors across the yMT gene.

Although these data provide evidence of the “core” genome of these organisms being identified, it could be that none of the factors and sequences that drive pathogenicity were present. Upon examination of the putative pathogens’ virulence plasmids, we found further evidence of a baseline level of pathogen presence. Specifically, for the stations with matches to *S. aureus*, we examined the coverage of the *mecA* gene, a gene associated with methicillin-resistant *Staphylococcus aureus* (MRSA) and nosocomial infections (Chambers and Deleo, 2009). We observed up to 323 coverage of the *mecA* gene (Figure 3A) but a wide range of coverage across all samples where it was present (0.23–323 coverage of the gene). We also examined the pMT1 plasmid of *Y. pestis*, which is a known virulence factor that can promote deep tissue invasion and acute infection symptoms (Lindler et al., 1998). We observed a similarly wide range of coverage from different samples (0.63–313) but consistent 203 coverage across the murine toxin (yMT) gene (Figure 3B) of the pMT1 plasmid, which is considered a virulence element for *Y. pestis* (Parkhill et al., 2001). We also used the SURPI algorithm to characterize these samples, which also predicted the presence of each of these pathogen-related organisms (Figure S5). Yet based on data from the CDC and HealthMap.org (http://www.healthmap.org/en/), which uses machine-learning algorithms to track all reported infections, there has not been a single reported case of *Y. pestis* in New York City since our collections began, indicating that these low-level pathogens, if truly present, are not likely active and causing disease in people.

**Figure 4:**
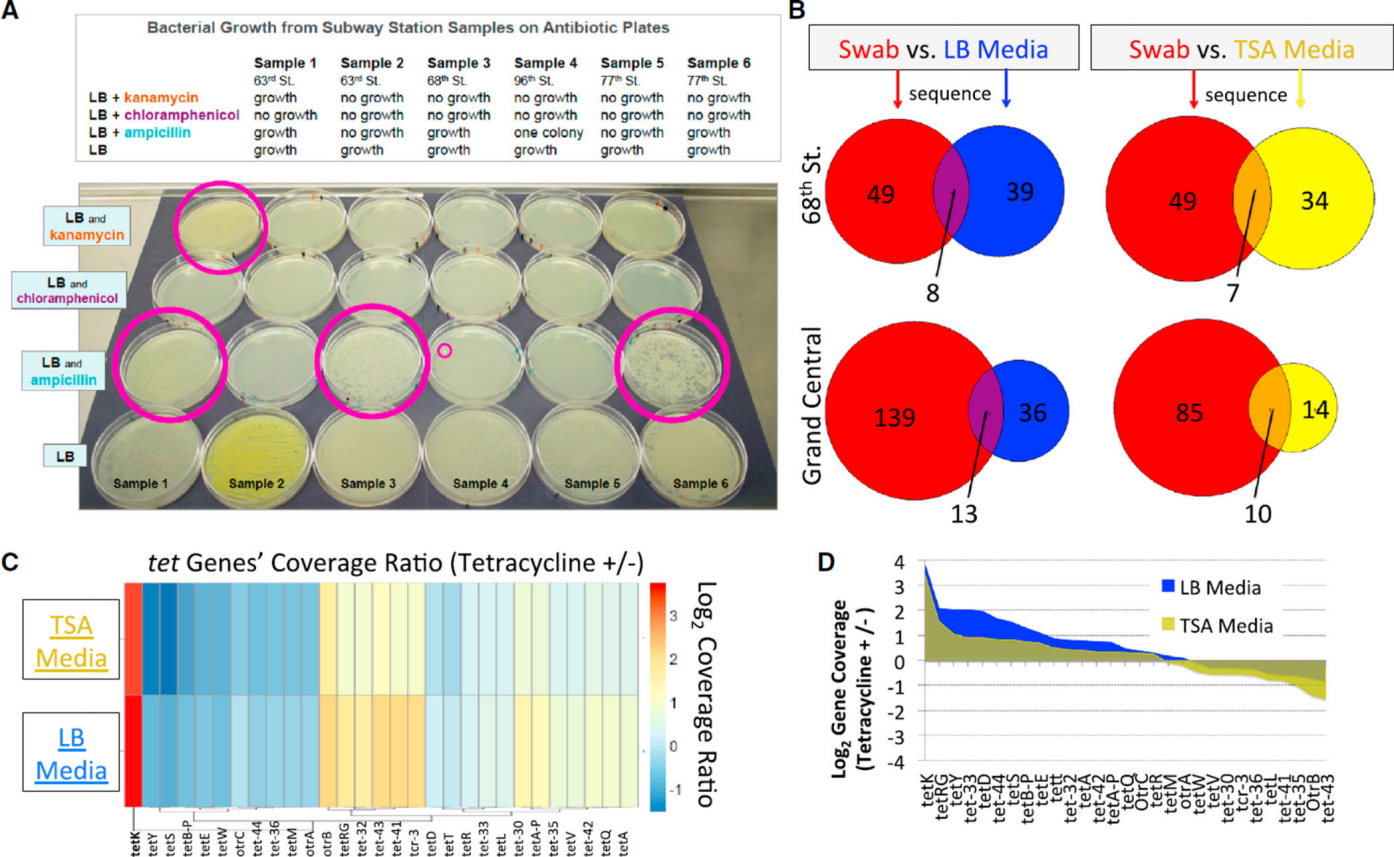
Live Strains of Antibiotic-Resistant Bacteria Cultured from City Surfaces (A) A single colony was plated across four plates for each site (above), then tested for three different antibiotics: kanamycin, chloramphenicol, and ampicillin. We found five plates (circled in pink) that showed growth even in the presence of antibiotics, including one site (far left) with resistance to two antibiotics, with growth in multiple rows. (B) Number of taxa found for the plain swab (red) versus the bacteria cultured and then sequenced from LB (blue) and TSA media (yellow). (C) The coverage of the tetracycline-resistance genes was calculated as the ratio of the Tet^+^ samples (treated with tetracycline) versus the original sample (non-treated, or Tet^−^), and the log_2_ ratio was plotted as a heat map (scale on left). (D) The distribution of coverage ratios for each tet gene for each of the cultured samples showed a greater coverage for the majority of *tet* genes in the Tet^+^ samples relative to the Tet^−^, untreated samples and a convergence on the *tetX* gene for samples on both media types.

To determine whether viable microorganisms could be cultured from the subway stations, we performed two experiments. First, we swabbed subway stations using the same protocol and then transferred the collection to four types of LB agar plates: one control and three with antibiotics (kanamycin, chloramphenicol, and ampicillin). We found that all plates (18/18) had viable bacteria that could be cultured on standard agar plates (Figure 4A). When we tested microorganisms cultured from swabs of the same stations, 28% (5/18) yielded colonies resistant to standard antibiotics (Figure 4A); one station produced a multi-drug-resistant culture. These results indicate, not surprisingly, that there are live bacterial communities present on the subway, but they also show that a substantive proportion of these possess some resistance to commonly used antibiotics.

We then performed a second culture experiment, combined with sequencing, to gauge the impact of medium type and to discern the genetic elements that may drive antibiotic resistance. We took samples from a subset of the same stations and cultured them on LB agar medium and Trytic Soy Agar (TSA) medium, while simultaneously testing the bacteria for resistance to tetracycline at two different temperatures (Table S5 and Experimental Procedures). We then sequenced the bacteria using the same methods as above, with taxa identified by BLAST and MetaPhlAn. We observed that sequence-based characterization of the samples consistently yielded an identification of more species than the culture-based methods (25%–380% increase), with an overall 20%–71% of the overlap between both methods (Figure 4B). We observed that the stations with the greater levels of human traffic (Grand Central, Times Square) had the greatest diversity of taxa (Table S5; Figure 4B), with a range of correlation of colony-forming units (CFUs) and daily passengers ranging from 0.66–0.72 (Pearson R^2^). In all cases, as expected, the application of tetracycline reduced the number of CFUs observed for each collection. Finally, we used the known antibiotic resistance genes from the Short Read Sequence Typing for Bacterial Pathogens (SRST2) database (Inouye et al., 2014) to examine the presence and dynamics of the tetracycline-resistance genes in our samples. We observed 29 of the known tetracy-cline-resistance genes across our cultures, and we then compared the overall coverage of each of these genes in the samples before and after tetracycline treatment (Figure 4C). The most significantly increased resistance gene, *tetK*, was present and significantly enriched relative to all other genes (t test, p = 0.003) across both types of media (Figure 4D); this gene is a known genetic driver for the tetracycline-resistance phenotype (Dutra et al., 2014).

### Microbial Diversity Can Define Stations and Surfaces

**Figure 5:**
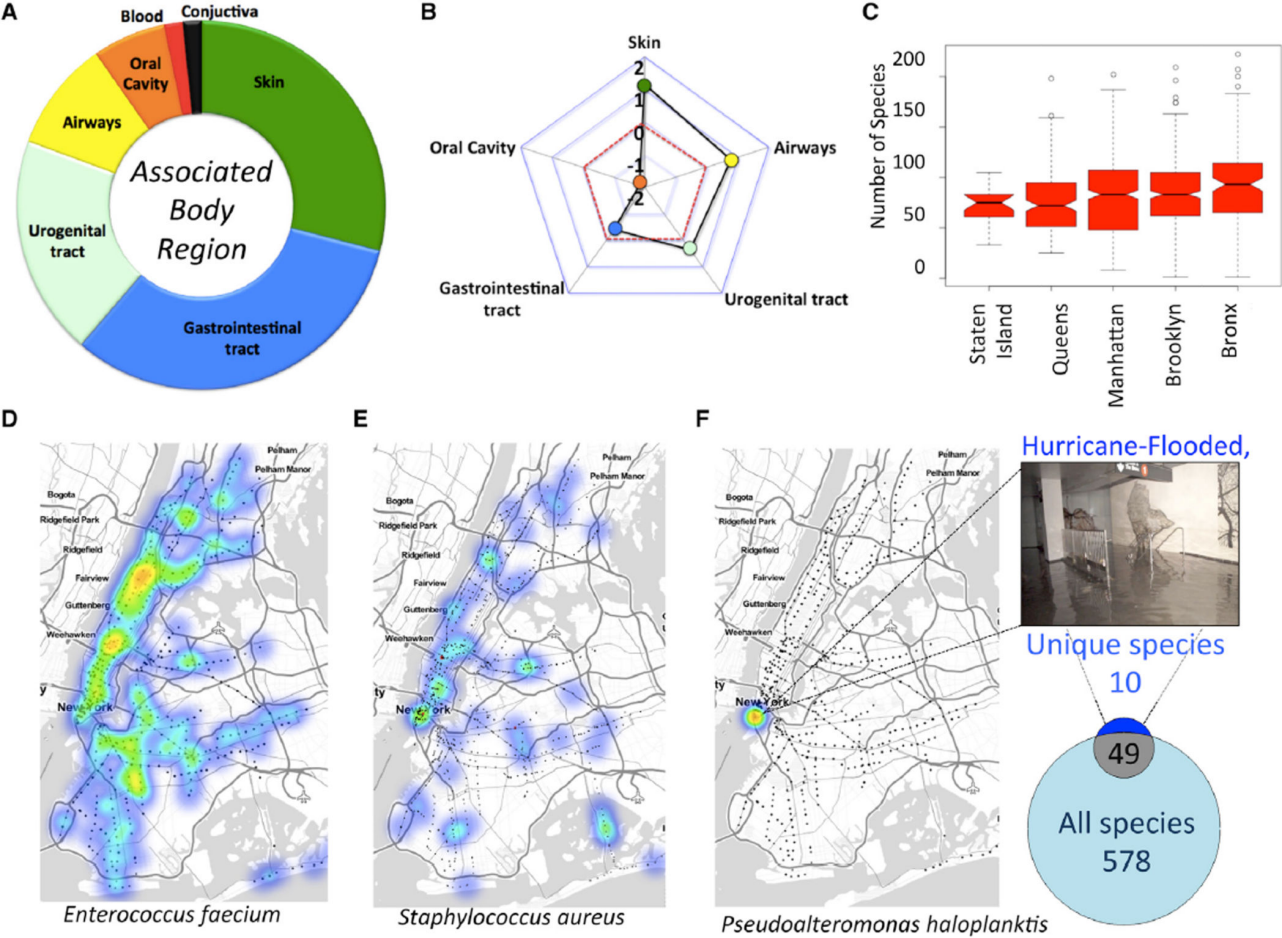
Taxa Diversity and Association with Human Body Areas Detected bacteria were annotated relative to the most commonly associated body part from the Human Microbiome Project (HMP) dataset. (A) Of the 67 PathoMap species that matched the HMP dataset, the proportions were greatest for the GI-tract (blue), skin (green), and urogenital tract (white). The entire circle represents 100% of the 67 species, and the sizes of each color represent the proportion of each type of bacteria. (B) To account for the database proportions from the HMP, we calculated the log_2_ of the observed versus expected numbers of species found for each category, which indicated that skin was the most predominant type of bacteria on the subway system. (C) Boxplot of the number of species found per borough. Middle line of each section shows the median, and the top and bottom of each box show the 75^th^ and 25^th^ percentiles, respectively. Notches show the significant difference between groups (95% confidence interval). (D and E) Heat maps of NYC showing the density for *Enterococcus faecium* (D) and *Staphylococcus aureus* (E). Small red dots indicate the presence of a fully re-sequenced *mecA* gene. (F) Analysis of a subway station (picture on top shows the station) flooded during Hurricane Sandy. The Venn Diagram compares the unique set of 10 species in the data from that station that did not appear in any other station or area of NYC, but 52 species overlapped with the set of 627 species present in the subway system.

To further catalog the types of bacteria that colonize the subway’s surfaces, we used the annotations from the Human Microbiome Project (HMP), which has assigned each bacterium to a primary area of the human body (see Experimental Procedures). Our data showed that the predominant species on the surfaces of the subway were associated with the skin, gastrointestinal tract (GI-tract), and urogenital tract (Figure 5). However, the HMP database has a different proportion of bacteria for each of these regions of the body, with a much higher number of known GI-tract bacteria (n = 371 species) versus the airways (n = 49). Thus, when calculating the enrichment of expected versus observed bacteria, based upon these normalized proportions, we found that the subway is most strongly associated with skin bacteria (8 expected versus 18 observed, a 2.3-fold enrichment). Thus, the subway’s microbiome is most highly enriched for skin (Figure 5B), including species like *Staphylococcus aureus* (Figure 5). Other enrichments included the airways (1.7-fold) and the urogenital tract (1.2-fold), whereas the under-represented categories were the GI-tract (−1.6-fold) and the oral cavity (−3.5-fold). This means that although some classes of bacteria, such as the GI-tract and *Enterococcus faecium*, may be abundant across the subway, these are actually lower than expected from known annotations, whereas the skin bacteria represent a strong enrichment from the baseline HMP data.

We next examined the distribution of global and unique taxa across the subway stations. We observed highly variable levels of concentrations for different species (Figures 5C–5F), and even between cumulative diversity at the borough level. Specifically, the Bronx showed the greatest level of bacterial diversity (Figure 5C), which was significantly higher than other boroughs (all p values < 0.001, ANOVA), whereas Brooklyn and Manhattan were more mid-range, and Staten Island held the lowest diversity. The station with the most unique bacteria was the South Ferry Station on the “1” subway line in Manhattan (Figure S6). This was the only station completely flooded during Hurricane Sandy in 2012, and it has been closed since that time. Notably, we observe ten unique species of bacteria that were present in the single flooded station and were not present in any of the other MTA stations or other samples (Figure 5E); by comparison, the next station with the most unique species had only four (Figure S5). The flooded station contained many species normally associated with cold marine environments, such as *Psychrobacter cryohalolentis, Pseudoalteromonas haloplanktis, Shewanella frigidimarina, Shewanella putrefaciens, Psychrobacter arcticus*, as well as several unclassified strains of *Carnobacterium, Cellulophaga*, *Flavobacterium*, and *Pseudoalteromonas*. Some of these species, like *Shewanella frigidimarina*, were previously assumed to be Antarctic species that are usually found associated with fish (Frolova et al., 2011). The data show how the walls and floors of the station still carry a “molecular echo” or microbiome aura (Lax et al., 2014) of the flooding of the station with cold ocean water.

To determine whether the marine signature of the South Ferry Station was a consequence of being coated in NYC’s waterways during the hurricane, we compared these data to 12 sites along the Gowanus Canal (GC) of Brooklyn, taking water samples and then processing, extracting, and sequencing the samples in the same fashion as above. We observed that the taxa unique to the hurricane-flooded, abandoned (AB) station were still distinct from those found in the Canal in Brooklyn (Figure S7). Although one sample (AB009) clustered with the GC samples, the majority of the samples clustered by the taxa of each site and showed distinct profiles. For example, the marine and Antarctic species of the South Ferry Station were not found in the GC samples, and the GC showed a unique enrichment for desulfobacter-and methanogen-related bacteria and archaea (Data Table 2; Figure S7), which may represent the industrial history of that site and its current status as a U.S. Environmental Protection Agency Superfund site.

### Dynamics and Functional Characterization of the Microbiome

**Figure 6:**
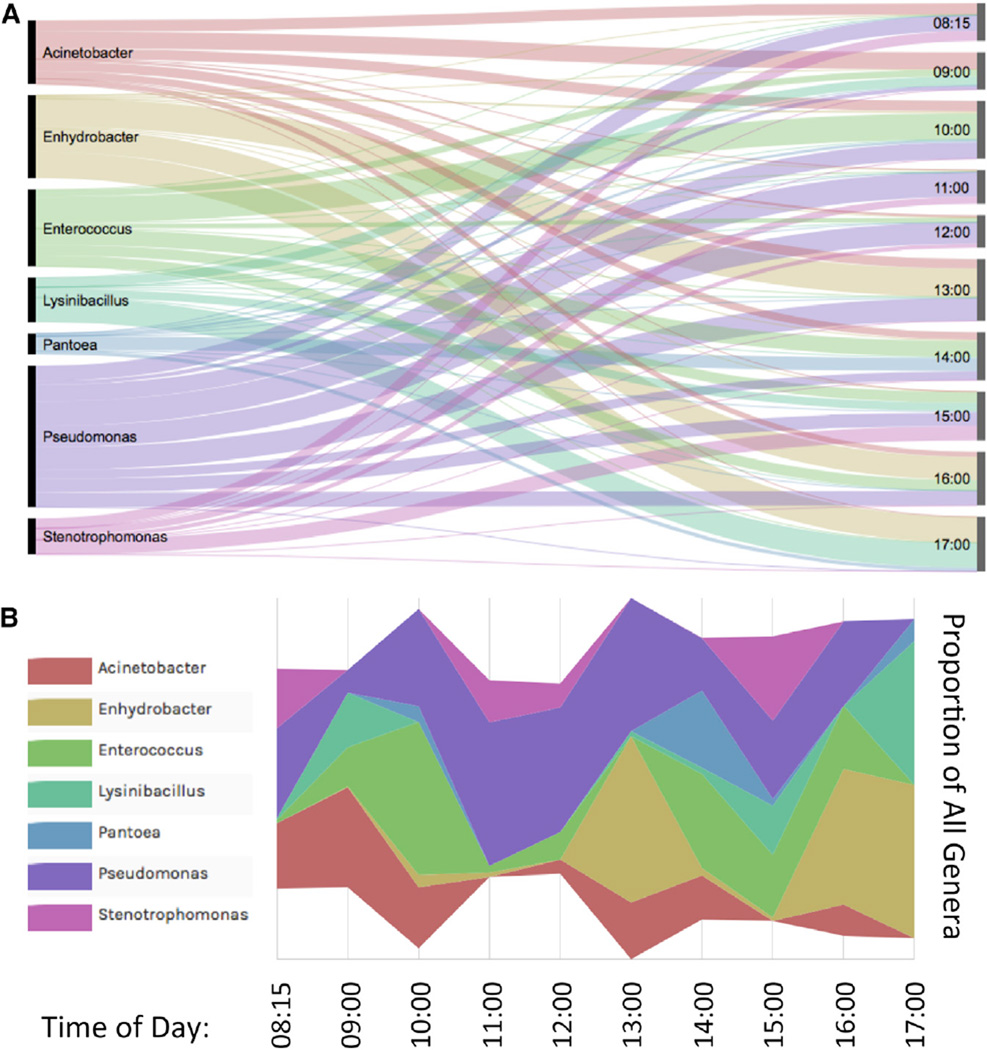
Hourly Dynamics of a Train Station Microbiome Analysis of samples collected at Penn Station on one day, compared at each hour. (A) The proportional distribution of taxa (left) to the proportion of their presence at a specific time (right). The thickness of each line is in linear proportion to the number of detected taxa. (B) Proportion of each bacterial taxa (by genus) at each time point. Each taxa is colored and labeled in-line according to the same schema as in (A). The maximum number of species (n = 64) was found at 13:00, and the minimum (n = 51) at 11:00, which is proportional to the width of the plot.

To gauge the persistence of a microbial signature at a station, we sampled one train station (Penn Station) in triplicate every hour on the hour during a weekday, then processed, sequenced, and analyzed the samples using the same procedures as for other samples. We found that certain taxa, such as *Pseudomonadaceae*, *Enterococcaceae*, and *Moraxellaceae*, are prevalent at every time point (Figure 6A). Yet a high degree of fluctuation was observed in some genera over the course of the day. For instance, *Pseudomadaceae* has its greatest abundance between 11:00 and 13:00, and *Moraxellaceae* was greatest at 17:00 at the end of the day. However, for the majority of families, the peaks greatly vary by the time of day, with low traces at the rest of the time intervals.

We next compared these data to public MTA data regarding the usage of turnstiles in the subway system at each station (http://web.mta.info/developers/turnstile.html), based on reported 8 hr increments, and correlated this to our DNA yield and overall taxa diversity. We found a slight trend for an increase in the amount of DNA collected over the course of the day (Figure S8), which matched the increasing number of riders at this station. However, neither of these trends were significantly associated with an increase in the total bacterial diversity at this one site (Figure 6). Rather, the dynamics of a single place on one station showed a consistent shifting of the taxa present (Figure 6B), with usually only 5%–10% of the taxa (especially for *Pseudomonas*) persisting as tens of passengers transit through the station.

Nevertheless, because the number of CFU counts from cultures showed a positive correlation with the number of riders (Table S5), we sought to expand this analysis beyond simply one station. We used 2010 U.S. Census data for NYC to calculate the overall degree of species diversity of a subway station and the population density of each area of the city. Overall, we found a low but positive correlation between the density of people living in an area and the degree of DNA diversity found at that site (R^2^ = 0.21, Figure S9A). Thus, this is consistent with a hypothesis that the density of people living in an area may contribute to a diverse surface-based microbiome. Moreover, when we examined the species diversity as a function of the ridership of the specific subway station, we also found a low but positive correlation (R^2^ = 0.20) between the number of commuters and the number of taxa found at a site (Figure S9B).

Finally, to characterize the functional properties of the bacterial and eukaryotic species identified on the subway, we performed additional 16S and 18S rRNA gene amplification and sequencing. First, we validated 23/29 eukaryotic species, including organisms like chickens, trichomonads, and spiders, by 18S rRNA gene sequencing (Figure S10). These results confirm the earlier BLAST results that showed the presence of a variety of insect species present on the subway, and we observed a median 0.63 correlation (R^2^ Pearson) between quantification levels from shotgun data versus 18S rRNA (Figure S10C). These data also expand the list of likely mammalian DNA left on the subway, which can arise from transit from other areas of the city (e.g., zoos, parks), leftover elements of food (beef and chicken meals), or animals and objects from people’s homes (dogs, cats, bags).

For four samples, we re-sequenced 16S rRNA gene amplicons (see Experimental Procedures), and analyzed the data with QIIME (Caporaso et al., 2010) and PICRUSt (Phylogenetic Investigation of Communities by Reconstruction of Unobserved States) (Langille et al., 2013), which utilizes the operational taxonomic units (OTUs) defined by known genes to annotate the putative metabolic and biological functions of a sample (Table S6). The top three OTUs for all tested samples were transporters, general function, and ABC transporters, with an enriched annotation from the KEGG pathway database for “environmental information processing, membrane transport, and transporters.” The largest other pathway enriched in these data was annotated as “unclassified, poorly characterized, and general function prediction only.” These annotations also show a strong enrichment of transporters and DNA replication and repair (including many species with radiation resistance or desiccation resistance phenotypes), which may indicate the inherent need for these bacteria to be continuously processing biological products from their human hosts, as well as the molecular tools needed for survival on primarily inert surfaces such as steel, glass, and plastic.

## DISCUSSION

Whereas previous metagenomic studies have focused on targeted areas in cities, this dataset represents a complete molecular portrait of the distribution of human and microbial diversity at a city-wide scale. Such data are critically important to ongoing efforts that are using DNA-based sequencing methods for health surveillance and potential disease detection (Tringe et al., 2008), as they define the baseline levels of potential pathogens along with normal flora (Blaser, 2014). Our data indicate that densely populated, highly trafficked areas of human transit show strong evidence of bacteria that are resistant to antibiotics and some presence of potentially pathogenic organisms. But, most importantly, these potentially infectious agents are not creating widespread sickness or disease. Instead, they likely represent normal co-habitants of a shared urban infrastructure, and they may even be essential to maintaining such an environment (Gilbert and Neufeld, 2014) and likely represent a normal, “healthy” metagenome profile of a city.

Indeed, these data indicate that the subway, in general, is primarily a safe surface. Although evidence of *B. anthracis, Y. pestis*, MRSA, and other CDC infectious agents was found on the subway system in multiple stations, the results do not suggest that the plague or anthrax is prevalent, nor do they suggest that NYC residents are at risk. According to the CDC, plague cases from 1970–2012 were heavily concentrated on the West Coast (http://www.cdc.gov/plague/maps/). Approximately seven human plague cases are reported a year, and none recently in NYC or anywhere near NYC, and these results match those present in HealthMap.org. This finding further supports the notion that humans have interacted (and potentially evolved) with their environment in such a way that even low levels of *Yersinia pestis* (plague) or *Bacillus anthracis* (anthrax) will not necessarily confer a risk of acquiring these pathogens.

The detection and classification of any putative pathogenic organism depends on many factors. These factors include the following: infective dosage, immune state of the hosts, route of transmission, other competitive species, informatics approaches to species identification, horizontal transfer (Smillie et al., 2011), bacterial methylome state and unique base modifications (Rasko et al., 2011), and other factors of microbial genome regulation. Notably, the evidence for these organisms came from multiple subway locations, was collected by different people, and was sequenced in two different facilities, and none of these organisms are studied in the laboratories where this research was conducted. As such, although the evidence is strong that these organisms were detected based on the current databases, it is always possible that improved bacterial annotations and newly completed genomes can move the “best-hit” evidence to a different species in the *Yersinia* or *Bacillus* genera, or a different genus altogether. Most importantly, none of these data indicate that these organisms are alive, and the fragments of bacterial DNA detected in these data may have arisen from sources other than humans (insects, rats, mice, or other mammals).

Recent work has shown that homes can create a specific microbiome profile or “aura” for families and that this profile travels with individuals (Lax et al., 2014). Yet, it was unknown how specific such a profile may be for mass-transit areas like subways. These data show that some events, such as a flooding event during a hurricane, can have a long-lasting impact on subway stations. Owing to the heavy rains of Hurricane (Superstorm) Sandy in 2012, the South Ferry Station was completely submerged in ocean water. Two years later, the majority of the bacteria from the South Ferry Station are still distinct from the rest of the entire subway system (Figure 5), and they mirror bacteria that are more commonly associated with fish species, marine environments, or very cold Antarctic environments; yet these species are still distinct from another waterway (Gowanus Canal) in Brooklyn. When the South Ferry station completely re-opens, it remains to be seen how long it will take for such a high-traffic urban area to be bio-remediated and normalized to mirror other stations, or if this unique profile of that station will persist long-term.

The rapid bacterial dynamics of Penn Station suggest that, even on an hourly basis, there is a vast bacterial ecology that is constantly shifting around commuters, which likely represents the diverse ecology of human urban populations (Gonzalez et al., 2012; Tyakht et al., 2013; Be et al., 2014). This diversity is confounded with the thousands of passengers traveling through the subway system, their personal microbial histories, station air flow, subway-cleaning frequencies, surface composition, and the particulars of this one site. Further high-resolution sampling will be required to discern the consistency of a station over a day, a month, or a year. To contextualize these results beyond NYC, matching protocols and methods will need to be applied in other cities’ public areas that represent other aspects of the built environment, such as subways, sewers, parks, and high-traffic subways; some of this work has started within the Meta-Sub project (http://www.metasub.org), which is creating these profiles across subways and cities around the world. Finally, additional positive controls are sorely needed for future sampling protocols, as is already done for clinical DNA and RNA sequencing (Munro et al., 2014; Li et al., 2014a, 2014b; SEQC/MAQC-III Consortium, 2014). This could include barcoded, synthetic, and titrated oligonucleotides being sprayed at regular intervals to account for the degradation, disturbance, and dissemination of DNA.

One notable result from these data was the conclusion that half of our high-quality sequence reads do not match any known organism, which is similar to the range reported in other studies (Yooseph et al., 2013) and demonstrates the large, unknown catalog of life directly beneath our fingertips that remains to be discovered and characterized. Because the majority of the DNA left on surfaces is bacterial, many of these unknown DNA fragments likely represent un-culturable species and strains of bacteria. Although different methods are needed to enrich for the metagenome of eukaryotes, we did observe a large catalog of potential eukaryotes on the subway (Data Table 1), and we speculate that their accurate detection is confounded both by the heterogeneity of the samples’ DNA as well as the simple fact that not all eukaryotic genomes have been sequenced. However, even at stringent frequencies, our rarefaction plots show that hundreds, to potentially thousands, of species may be present in the subway (Figure S11). These taxa found in the subway also match many of the same species found in the air (Table S7). The top-ranking eukaryotic species (Table 1) include organisms that are not often seen in the subway, such as mountain pine beetles and Mediterranean fruit flies; these likely represent the closest fully sequenced organisms present in NCBI and other genome sequence databases. This work highlights the ongoing need for robust eukaryotic genome assemblies to be completed, such as the Genome 10K project (https://genome10k.soe.ucsc.edu/) and the insect i5K project (http://www.arthropodgenomes.org/wiki/i5K). Also, there have been documented cases of lateral gene transfer of bacterial genes into *Drosophila* or other insect hosts (Klasson et al., 2014), as well as contaminants of bacteria present in genome assemblies (Salzberg et al., 2005), both of which may impact the interpretation of these results across eukaryotic and other taxa.

Interestingly, such metagenomics profiling of a city, as shown here, could facilitate new forensic applications that use station-specific taxa (Figure 5) and the distribution of ancestry-informative markers from shotgun genomic DNA (Figure 2), just as genetic markers informative of human ancestry can reveal the likely origin of a person’s birth (Novembre et al., 2008). For example, the bottom of a person’s shoe might represent the “genetic history” of that person’s daily or weekly travels, and the molecular data can reveal the proportion of unique genetic markers and potentially define the geospatial-genetic history of a person in a city, as well as his or her pathogen risk or threat. These applications of public genetic data create potentially ambiguous ethical situations, whereby one’s metagenome may hold clues about historical, geospatial-genetic history, which then reduce one’s expectation of privacy. But they also could provide new forensic tools and methods for criminal justice and also new mechanisms for disease and threat surveillance that are needed in increasingly urbanized human societies.

Such “big data” could even be combined with a complete human genome to predict a person’s degree of baseline immunological protection/risk, combined with a characterization of the dynamic antibodies and IgG variable regions in the person (immunomics) relative to the microbial alleles/strains present in a city. Ideally, these data and methods can be utilized for improved monitoring of microbial biology vis-à-vis human biology, in the built environment of mass transit. For this to occur, however, other cities’ baseline pathogen and microbial profiles will be needed, to help contextualize all of these data, concomitant with improved sequencing lengths and expanded reference databases. Finally, further development of faster, even real-time, characterization of the dynamics of the urban metagenome and mass-transit systems can enable a more nimble response time to any perturbations of these systems, which could potentially impact the lives of millions of people each day and billions of people each year.

## EXPERIMENTAL PROCEDURES

### Sample Collection

The entire NYC MTA subway system, a total of 468 stations, was swabbed in triplicate over the course of the summer of 2013 and some additional samples taken for culturing and testing and in response to reviewers in 2014. Two surfaces were swabbed in each station, and one surface was swabbed within the train. Samples were collected from turnstiles and emergency exits, Metro Card kiosks, wooden and metal benches, stairwell handrails, and trashcans. The turnstiles and kiosks were prioritized at each station due to the level of human-surface interaction at these particular sites. In the train, the doors, poles, handrails, and seats were swabbed.

Samples were collected using Copan Liquid Amies Elution Swab 481C, a nylon-flocked swab with a 1 ml transport medium. The transport medium maintains a pH of 7.0 ± 0.5 and consists of sodium chloride, potassium chloride, calcium chloride, magnesium chloride, monopotassium phosphate, disodium phosphate, sodium thioglycollate, and distilled water (Amies, 1967). After a surface was sampled, the swab was immediately placed into the collection tube, coming into contact with the transport medium; samples were then stored in a −80°C freezer once returned to the laboratory.

A mobile application (app) for iOS and Android systems was developed in collaboration with GIS Cloud Mobile Data Collection (MDC) to map the data points according to their geographical locations. When using the GIS Cloud app, data fields to input included a sampleID, place, surface, traffic level, notes, and an option to take a picture, and the app automatically adds a time stamp for each submission (Figure S12). The app has been expanded to include swabbing of other surfaces, including buses, taxis, parks, and airports. All data points are accessible to view via pathomap.giscloud.com.

### DNA Extraction

Samples were brought out of the −80°C freezer to thaw to room temperature. DNA was extracted using the MoBio Powersoil DNA isolation kit (as seen in Qin et al., 2010 and also http://www.mobio.com/soil-dna-isolation/powersoil-dna-isolation-kit.html). Using the reagents from the kit, the sample’s cells were lysed, freeing the DNA and other contents. The other inorganic material was precipitated out. Using a concentrated salt solution, the DNA readily bound to the silica membrane of the kit’s spin filters. An ethanol wash helped further clean and purify the DNA. Following the MoBio protocol, the 50 µl eluent was further purified by introducing 100 µl (2:1 ratio) of Agencourt AMPure XP magnetic beads. Samples were left to incubate at 25°C for 15 min and placed on an Invitrogen magnetic separation rack (MagnaRack) for 5 min. The DNA binds to the beads, and the supernatant is discarded. While the tubes were on the MagnaRack, 700 µl of 80% ethanol was added to the beads to wash off any remaining impurities. The ethanol was removed, and beads were left to dry. Finally, 10 µl of an elution buffer was added to purify the DNA, and 9 µl of the eluent was removed with 1 µl going toward QuBit quantification. Using a Qubit 2.0 fluorometer and the high-sensitivity kit (DNA HS standards, dsDNA HS buffer, and HS dye), we quantified each sample’s DNA. The parameters of the QuBit were set for ng/µl, and the value from the device was then multiplied by 8 µl for the total yield of the sample in ng.

### Illumina and QIAGEN Library Preparation

DNA fractions were prepared into sequencing libraries according to manufacturer’s standard protocols, using the TruSeq Nano DNA library preparation protocols (FC-121–4001). A subset of our samples (Culture 01–12 and other test samples) was also prepared using the QIAGEN Gene Reader DNA Library Prep I Kit (cat. no. 180984). Briefly, this involved Covaris fragmentation to ∼500 nt, bead cleanup to remove small fragments (<200), A-tailing, adaptor ligation, PCR amplification, bead-based library size selection, and cleanup again. Fragments were then visualized on a BioAnalyzer 2100 to ensure libraries were within the range of 450–650 bp.

### Sequencing

Raw data from four flowcells of the HiS eq 2500 machines using HiSeq (v4) SBS chemistry were processed using the Illumina RTA software and CASAVA 1.8.2, and then all samples checked for standard CASAVA QC parameters (all reads pass filter). Specifically, all samples had high (>Q20) quality values at the median base, low % alignment to PhiX (<1%), and similar insert size (550 ± SD of 70 bp).

### Sequence and Taxa Characterization

All reads were first quality trimmed with the FASTX toolkit (http://hannonlab.cshl.edu/fastx_toolkit/) to ensure 99% base-level accuracy (Q20). Cleaned reads were then aligned with MegaBLAST (Wolfsberg and Madden, 2001) (see Experimental Procedures) to search for a match to any organism in the full NCBI NT/NR database. The MegaBLAST output for one read often returns multiple hits to sequences from different taxa, so we assigned each read to a single “best” taxon using the LCA algorithm established by MEGAN (Huson et al., 2007). For example, the species *Salmonella enterica* and the species *Salmonella bongori* may have ambiguous reads that match both species, but the LCA (genus *Salmonella*) can have sequences unique to that genus, which is then the assigned taxa. To further classify bacterial and viral sequences (see Experimental Procedures), we also analyzed all samples with MetaPhlAn 2.0 (Segata et al., 2012), and for specific pathogens, we also used SURPI (Naccache et al., 2014) and the BWA (see below) (Li and Durbin, 2010).

MetaPhlAn version (v2.0) was used to study the microbial populations on the subway surfaces. FASTQ files from sequencing were run through MetaPhlAn (see command in Supplemental Experimental Procedures), and the output file (.bt2.out) outlined the abundance of various bacterial organisms to the species level.

### BWA Alignments

BWA was used to align sample sequences against several reference genomes, including the virulence plasmids. Standard genome processing of the genomes was performed with BWA (version 7.10, http://bio-bwa.sourceforge.net/bwa.shtml), which includes building a burrows-wheeler transformation of the reference genome, performing an alignment (aln ref.fa short_read.fq > aln_sa.sai), and then converting the suffix array into genome coordinates and a SAM file (sampe ref.fa aln_sa1.sai aln_sa2.sai read1.fq read2.fq > aln-pe.sam). SAM tools version 1.19 (http://samtools.sourceforge.net/samtools.shtml) was also used to call genetic variants (sam tools mpile up -C50 -gf re-f.fasta -r chr3:1,000–2,000 in1.bam in2.bam) compared to the reference genome. All commands and scripts used are detailed in the (Supplemental Experimental Procedures and are the exact shell commands (.sh files) we used to process the raw data.

To further investigate results of potential pathogenic bacteria found on the subway, each sample’s sequences were compared to the virulent plasmid(s)’s sequence. Using the National Select Agent Registry (NSAR) select agents and toxins list (notably, CDC Tier 1 agents) and the PATRIC database, a list of pathogenic organisms was determined and cross-referenced to results from Meta-Phlan and BLAST. To verify these results, sequences of virulent plasmids of the various agents were found on GenBank, and using BWA and the Integrative Genomic Viewer (IGV), the sample was compared to the reference sequence.

### Human Body-Part Association with Species

Species were matched to the top-associated human body part from the Human Microbiome Project’s (HMP) public database, located here: http://www.hmpdacc.org/HMRGD/healthy/. We used the top-ranked species for each area of the body listed in the HMP dataset.

### Bacterial Cultures, Collection, and Sequencing

Swab samples were collected from eight NYC subway locations to determine whether bacteria could be cultured from turnstiles, and whether these culturable bacteria would grow in the presence of tetracycline. Collection locations within the subway system were selected based on the intensity of human use to determine whether the concentration of culturable bacteria would increase with the level of human traffic. Four turnstiles from “low-traffic” stations (68th St station, 5th Ave/53rd St Station, 77th St Station, and 8th Ave/50th St Station) and four turnstiles from “high-traffic” stations (from two separate locations within both 42nd St Grand Central Station and 42nd St Times Square Station) were sampled in March 2014 (Table S4). Immediately prior to sample collection, swabs (Elution Swabs; Copan Diagnostics) were dipped into the 1 ml of sterile Amies transport media supplied with the swab kit, as pre-moistening of swabs has been shown to improve bacterial recovery from environmental surfaces. Two arms of each turnstile were swabbed at a constant speed for a total of 1 min, and one individual performed all swab sampling in order to standardize sampling effort. Swabs were then sealed within the sterile polypropylene tubes supplied with the ESwab kit, packed into a cooler, transported to the laboratory, and stored at 4°C for less than 24 hr before processing.

Cultivation of each sample began by briefly vortexing swabs to resuspend cells in the transport media prior to creation of 0–3 10-fold dilutions in auto-claved and 0.2 µm filter sterilized 25% Ringers Solution (Oxoid). One hundred microliters of each dilution was spread on Luria Broth Agar (LB; Difco) and Trytic Soy Agar (TSA; Difco) media, each with and without tetracycline (10 mg/l) added. Control plates, spread with only sterile Ringers solution, were used as a method blank and processed in parallel with the swab samples. Enumeration of CFUs occurred after replicate plates were incubated at 28°C and 37°C for 5 days. The number of CFUs was then normalized to the concentration within the original 1 ml of transport media and reported as CFUs per 1 min of standardized swabbing effort, to allow a relative comparison among subway swab samples. Following incubation and enumeration, cells were harvested by pippetting 2 ml of sterile water (Hyclone) onto each plate and using a sterile spreader to scrape colonies from the media surface into a suspension. The cell suspension was transferred to a sterile tube, and DNA from this cell suspension was extracted (see above) to allow NGS characterization of the cultivated bacterial assemblage.

### MegaBLAST-LCA Pipeline

The MegaBLAST-LCA pipeline consisted of five steps explained in detail below. (1) Paired-end reads were prepared for BLAST by trimming, filtering on quality scores, and converting to unpaired FASTA sequences. (2) Prepared reads were searched for in the NCBI NT database using MegaBLAST (default parameters). (3) MegaBLAST hits were filtered such that short and low-scoring hits were ignored in subsequent analysis. (4) Reads with MegaBLAST hits to multiple taxa were assigned to the LCA taxa in the NCBI Taxonomy using the MEGAN algorithm. For example, hits to multiple species of the same genus are assigned to the common genus by the LCA algorithm. (5) Finally, for each sample, the total number of reads assigned to each taxon were counted. We validated our MegaBLAST-LCA pipeline on a mock community of 11 bacterial species (see Tables S2 and S3).

### Preparing Reads for MegaBLAST

The leading and trailing 10 bp were trimmed from the 100 bp reads to remove low-quality regions. Trimmed reads with more than 10 bases with quality scores less than 20 were removed. Only one read from each pair was analyzed further because MegaBLAST does not accommodate paired sequences.

### Removal of Low-Scoring and Short-Length MegaBLAST Hits

MegaBLAST hits covering less than 65 bp of the 80 bp query sequence were removed. We further filtered MegaBLAST hits following the recipe of the MEGAN software. We required a min-score of 60 and a top percent of 10. Thus, hits with a MegaBLAST bitscore lower than 60 were ignored, and hits that were not within 10 percent of the best bitscore were ignored. Finally, we implemented a win-score of 100, requiring that, for a given query, if at least one hit had a bitscore greater than 100, hits with bitscores less than 100 were ignored. See the MEGAN paper for further explanation (Huson et al., 2007).

### LCA Algorithm

LCA was introduced as a bioinformatics method for estimating the taxonomic composition of a metagenomic DNA sample (Huson et al., 2007). MEGAN is a popular implementation of the LCA algorithm by the same authors. LCA is a very simple algorithm. Given a taxonomic tree (e.g., the NCBI Taxonomy) and a set of nodes in the tree (e.g., a few species), the LCA is identified by back-tracing from each node in the set until convergence at a single node— the LCA. We implemented the simple LCA algorithm following previously established methods (Huson et al., 2007).

### Positive Control

We used a positive control sample from the Metagenomics Research Group (MRG) of the ABRF (Association of Biomolecular Resource Facilities), and the control sample contained 11, and only 11, known bacteria that were sequenced with 150 3 150 paired-end reads on an Illumina His eq 2500 (v3). We used this sample to establish a minimum threshold for calling a species present (Figure S2 and Tables S1 and S2) from both BLAST and MetaPhlAn, which enabled us to estimate 99% specificity and 91% sensitivity at the genus level for MetaPhlAn. For BLAST, we observed 99.99% specificity and 100% sensitivity. To ensure robust analysis, we focus only those species found by both methods at these thresholds (normalized MetaPhlAn abundance of 0.01 and 0.1% of BLAST reads). This corresponds to an average minimum of 3,000 paired-end reads for each species. These NARG samples are also present in our SRA submission.

### Negative Control

In conjunction with the positive control, we had a subset of samples designated as negative controls. These swabs were taken out of their package and immediately placed in the collection tube, being exposed in the environment for no more than 1 s. The swabs were extracted following the same protocol as all the other samples. There were a total of 51 control blank samples collected, and 13 were extracted. The DNA yield was consistently found to be undetectable by Qubit (<0.05 ng/ml) for all samples. These data indicate that the DNA we are studying is collected from the environment and surfaces we swab and not from any other sources like the ESwab solution or MoBio Powersoil kit.

### Geospatial Image Segmentation

We used the Berkeley Image Segmentation Algorithm at http://www.imageseg.com/ to characterize the sub-sections and regions of the demographic map. The raw image was uploaded onto the online site and processed using a threshold of 40, shape rate of 0.6, and compactness rate of 0.2

### Ancestry Analysis Methods

#### Dataset Preparation

We have used two different methods in our ancestry analysis: Ancestry Mapper and Admixture (below). Both methods use a set of references that we have obtained by merging the genotypes from each PathoMap sample with the phase 2 whole-genome of the 1000 Genomes Projects, build hg19 (ref to 1000 genomes). In this manner, each PathoMap sample is included in a table of genotypes with each population (n), including the following: Yoruba (87), Luhya (96), African American (61), Puerto Rican (53), Spanish (14), Tuscan (98), Northern European Ancestry-Utah (82), British (88), Finnish (92), Han-Chinese (100), Han-Beijing (96), Japanese (89), Colombian (60), and Mexican (66).We merged the PathoMap VCFs with the file 00-All.vcf.gz, which provides a comprehensive report of short human variations formatted in VCF (http://www.ncbi.nlm.nih.gov/variation/docs/human_variation_vcf/#all-00); in this manner, we filtered for each PathoMap the SNPs that were useful in ancestry analysis. We then proceeded to merge this file with the VCFs from the 1000 genomes. We used VCF-tools and the commands VCF-merge and VCF-isec. We proceeded to merge the 1000 genomes by chromosome, and used a tped as output. The 23 tpeds were then merged using plink (Purcell et al., 2007).

#### Ancestry Mapper

Ancestry Mapper (Magalhães et al., 2012) calculates the genetic distance to a set of population references and provides a reference system to which every sample can be placed. Because it relates to a fixed set of references, it is less dependent on the context of the other samples in the dataset. It is a method suited to this problem, as the PathoMap samples do not have the same set of genotypes, hence each one has to be analyzed on its own. The references for Ancestry Mapper were calculated as the consensus of the individuals of each 1000-genomes population, and the genetic distance to each population was calculated by the euclidean distance. The Ancestry Mapper Ids (AMIds) were derived such that the most similar population got an index of 100 and the lowest an index of 0; AMIds are biologically meaningful as they relate to well-established populations. As positive controls, we calculated AMIds for each of the 1000-genome samples included in each PathoMap set of SNPs; they all correspond to what would be expected, i.e., Yoruba individuals got AMIds of 100 for the Yoruba reference and 0 for the Mexican sample; conversely, for Mexican individuals, the AMId for Yoruba was 0, with AMIds for Mexicans 100. It is worth pointing out that there is no 1000-genomes population that would correspond to a genetically homogeneous Amerindean population; we have used the Mexican population as a proxy for such population. Ancestry Mapper is available as an R package from CRAN (Magalhães et al., 2012).

#### Admixture

Admixture is a model-based ancestry estimation that directly seeks the ancestral clusters in the data (Alexander et al., 2009). Admixture models the probability of the observed genotypes to belong to ancestry proportions. We used Admixture on each set of PathoMap and 1000-genomes individuals and assumed the number of ancestral populations (K) to be 4; these ancestral populations correspond to African, Indo-european, Asian, and Amerindian. We verified that the 1000-genomes individuals were indeed assigned very high values for their corresponding ancestral populations (e.g., all African individuals were assigned very high values for an ancestral population that we inferred to be African). We took the values that were assigned to the PathoMap individual to correspond to their main ancestry components.

#### Software

We used Plink 1.9 (http://pngu.mgh.harvard.edu/~purcell/plink/plink2.shtml), VCFtools (http://vcftools.sourceforge.net/downloads.html), Admixture (https://www.genetics.ucla.edu/software/admixture/download.html), Ancestry Mapper (R package available at CRAN), and a series of shell scripts (Supplemental Experimental Procedures).

#### Reference Data

Please see 1000 Genomes whole genomes (http://www.1000genomes.org/data).

### 18S Validation

#### Sequencing and Library Prep

The protocol used for amplification and sequencing of the V9 region of the 18S rRNA gene is based off the 18S Illumina amplification protocol detailed on the Earth Microbiome Website (http://www.earthmicrobiome.org) (Gilbert et al., 2010). Briefly, PCR amplification of the V9 region was done in triplicate, cleaned, visualized as above, pooled following the EMP protocol, and sequenced on an Illumina Miseq with 2 × 100 chemistry (v3) with a 10% PhiX spike-in.

#### 18S Data Analysis

All data analysis and quality filtering were done following the QIIME pipeline (Caporaso et al., 2010). Paired-end reads were joined using fastq-join (Aronesty, 2011) with a minimum overlap of 10 bp, and only joined sequences were used for further analysis. Joined reads were de-multiplexed and quality filtered using the default parameters of split_libraries.py in QIIME. Additionally, Usearch (Edgar, 2010) version 5.2 was used to screen sequences for chimeras and singletons and cluster reads in to OTUs with a 97% similarity threshold following the de-novo protocol. Taxonomy was assigned using the SILVA database (Quast et al., 2013) version 111 no ambiguious base file reference database and UCLUST within QIIME. The resulting OTUs were filtered to exclude bacteria and archaea, and downstream diversity analyses used data rarefied to the lowest amount of sequences per sample (3,385). This left 551 OTUs from four samples.

### 16S Data Analysis

16S analysis followed the same steps as 18S; however, closed reference OTUs were picked with Usearch against the Green Genes database (DeSantis et al., 2006).

## Supplementary Material

suppl

## Figures and Tables

**Table 1:** Summary of Top Taxa Per Kingdom

Bacteria				Virus/Phages			
No.	Genus	Species	NCBI Taxa-ID	No.	Genus	Species	NCBI Taxa-ID
1,224	*Pseudomonas*	*stutzeri*	316	74	*Enterobacteria* phage	phiX174	374840

1,007	*Stenotrophomonas*	*maltophilia*	40324	28	*Epsilon15likevirus*	unknown	unknown

939	*Enterobacter*	*cloacae*	550	13	*Erwinia* phage	ENT90	947843

728	*Acinetobacter*	*radioresistans*	40216	12	*Enterobacteria* phage	HK97	37554

675	*Acinetobacter*	*nosocomialis*	106654	10	*Stenotrophomonas* phage	phiSMA7	1343494

555	*Lysinibacillus*	*sphaericus*	1421	9	*Staphylcoccus* phage	PVL	71366

544	*Enterococcus*	*casseliflavus*	37734	7	*Enterobacteria* phage	mEp235	1147150

460	*Brevundimonas*	*diminuta*	293	6	*Lactococcus* phage	ul36	374525

428	*Acinetobacter*	*lwoffii*	28090	6	*Stenotrophomonas* phage	phiSMA9	334856

427	*Bacillus*	*cereus*	1396	4	*Enterococcus* phage	phiFL3A	673837

This table shows the most abundant species (with the corresponding NCBI Taxa-ID) by kingdom and the number of samples in which these species were detected.

## Highlights

- Almost half of all DNA present on the subway’s surfaces matches no known organism.
- Hundreds of species of bacteria are in the subway, mostly harmless. More riders bring more diversity.
- One station flooded during Hurricane Sandy still resembles a marine environment.
- Human allele frequencies in DNA on surfaces can mirror US Census data.
