# Supplementary material for: Geospatial Resolution of Human and Bacterial Diversity with City-Scale Metagenomics
Source: Cell Syst. Author manuscript; Available in PMC 2016 Jul 29. (PMC4651444; doi:10.1016/j.cels.2015.01.001)
Supplement: suppl [file NIHMS662921-supplement-suppl.docx]

**Supplemental Figures**

**Figure S1 – DNA Yield per sample, by Type, in relation to Figure 1.** Data across all 1,469 samples showed an average of 185ng collected, even across a wide range of surface types (Turnstile, Bench, Kiosk, Garbage, Stairway Railing). This was ensured by a 3-minute swabbing protocol over a surface area of about one square foot.

**Figure S2 – Analysis of Positive Control Sample, in relation to Figure 1.** We used a sample sequenced from the Association of Biomolecular Resource Facilities (ABRF) Metagenomics Research Group (MRG) that contained 11 known species: (A) *Bacillus cereus*, (B) *Enterococcus faecalis*, (C) *Raoutella terrigena*, (D) *Bacillus megaterium*, (E) *Micrococcus luteus*, (F) *Pseudomonas aeruginosa,* (G) *Rhodospirillum rubra*, (H) *Sporosarcina ureae*, (I) *Streptomyces griseus*, (J) *Staphylococcus epidermidis*, (K) *Enterobacter aerogenes*. (M) After mixing, the expected proportions of DNA were near 9% for all species, but a larger range included 23% for *Staphylococcus epidermidis* and *Streptomyces griseus* (1%). (N) Measurements of the sequenced sample from MetaPhlAn and BLAST showed that BLAST detected all 11 species, whereas MetaPhlAn detected 9 species (as expected, since two species were not in the MetaPhlAn 2.0 database: *Sporosarcina ureae*).

**Figure S3 – True positive rate (TPR) and False Positive Rate (FPR) for two analysis pipelines**. We used both MetaPhlAn and BLAST-LCA for the analysis of the shotgun metagenomic sequence data, and we set our thresholds to ensure 99% TPR and an estimated 0.1% and 3% FPR (upper right point in both pipelines).

**Figure S4 – Geospatial Partition the NYC 2010 U.S. Census Map, in relation to Figure 2.** We used the geospatial image analysis software^18^ from the Berkeley Image Segmentation (BIS) Group to define areas of NYC with demographic homogeneity, We then compared these areas to the allele frequencies found in the subway samples that matched SNPs from the 1000 genomes project. We used the online interface to the segmentation software at <http://www.imageseg.com/>.

**Figure S5 – SURPI output of predicted pathogens, in relation to Figure 3.** We used the Sequence-based Ultra-Rapid Pathogen Identification (SUPRI) tool to provide additional evidence of Y. pestis from our dataset, which confirmed the presence of *S. aureus* and *Y. pestis* for all the samples found in MetaPhlAn and BLAST. Taxa are shown on the x-axis and the heatmap shows the proportion of reads supporting each species. All species required a minimum of 400 reads support for each bacteria.

**Figure S6 – Number of Unique Species Per Station, in relation to Figure 4.** We used the number of unique species found per station and plotted proportionally sized circles for each station to highlight the stations with the most unique numbers taxa. These data highlight the station impacted by Hurricane Sandy as the most unique.

**Figure S7 – Normalized MetaPhlAn Abundance of water-based and marine-associated samples, in relation to Figure 4.** Each of the samples from the floors, walls, and railings of the South Ferry station (abandoned, listed as AB) were clustered with the 12 samples collected from the water and shores of the Gowanus Canal (GC samples). All samples except one clustered by their origin. Normalized MetaPhlAn abundance is listed on the scale on the right (100 highest in red, 0 in blue as the lowest).

**Figure S8 – Hourly dynamics of a train station microbiome, in relation to Figure 6**. We plotted the number of bacteria taxa (left axis, blue bars), the DNA yield (in nanograms, ng, right-axis), and the number of riders per turnstile at this part of Penn Station (right axis, red dots) as a function of time. There was an upward trend of the number of riders and DNA collected, but not for the taxa diversity.

**Figure S9 – Population density, subway ridership, and species diversity, in relation to Figure 6**. (A) We plotted the bacterial species’ phylogenetic diversity (using Faith’s Index) for each station as a function of the population density for each area of New York City (y-axis, 1km radius). We observed a low, but overall positive correlation between the density of people living in an area and the degree of diversity found at that site (R^2^=0.21). (B) We then sough to characterize the number of distinct species found at each subway station as a function of the mean weekly ridership of that station, as reported by the MTA, and we also found a low, but positive correlation (R^2^=0.20).

**Figure S10 – 18S and 16S rRNA gene validation of species, in relation to Table 1 and Figure 6.** We used 18S and 16S primers from the Carlton lab at NYU and QIIME to detect various species found by our shotgun sequencing methods (A) We observed 23/29 of them confirmed, although the proportions were different between the data sets. (B) For bacterial content, we observed higher concordance of abudnace for some taxa (here, *Brevundimonas*), and (C) overall a median correlation of abundance between 16S and BLAST/MetaPhlAN output from all taxa identified in both methods.

**
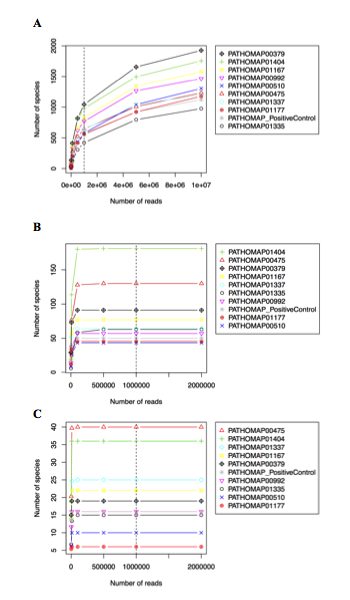
**

**Figure S11 -** **Rarefaction curves, in relation to Figure 6 and Table 1.** (A) The total number of species identified by MegaBLAST-LCA as a function of the number of randomly sampled reads. Data points are the mean value in three random replicates. Dotted vertical line marks one million reads. (A) False positive species identifications are substantial based on the results for PATHOMAP_PositiveControl, a mock community of 11 bacterial species. (B) As a crude measure to decrease false positives, only species identified at greater than 0.001 reads per million are included. Sampling more than one million random reads does not markedly increase the number of species identified using this abundance criteria. (C) To decrease false positives, only species identified at greater than 0.01% of total reads are included, which achieves the correct order of magnitude for the mock community. Sampling more than one million random reads does not markedly increase the number of species identified. Overall, identifying additional low abundance species incurs the cost of low specificity.

**Figure S12- Current collection sets and mobile app for data collection, in relation to Figure 1.** Our online maps (A,B,C,D) show all the sites that have been collected for the Pathomap project, using methods described herein. The bottom panels (E-H) show screen shots of our auto-fill mobile application (iOS and Android) for cataloguing the samples as they are collected. More details are also provided in the Volunteer Instructions .pdf in supplementary materials.

**Supplemental Tables**

| **Supplemental Table 1: Sensitivity and Specificity of Identification Methods by Species** | | | | | | |
| --- | --- | --- | --- | --- | --- | --- |
|  |  |  |  |  |  |  |
| MetaPhlAn (Normalized Proportion Value) - By Species | | | | | | |
| Threshold | Sensitivity (TP/(TP+FN)) | True Positives | False Negatives | Specificity (TN/(FP+TN) | True Negatives | False Positives |
| 10 | 27.3% | 3 | 8 | 100.0% | 16904 | 0 |
| 1 | 81.8% | 9 | 2 | 100.0% | 16903 | 1 |
| 0.1 | 81.8% | 9 | 2 | 99.9% | 16894 | 10 |
| 0.01 | 81.8% | 9 | 2 | 99.9% | 16884 | 20 |
| 0.001 | 81.8% | 9 | 2 | 99.9% | 16882 | 22 |
| 0.0001 | 81.8% | 9 | 2 | 99.9% | 16882 | 22 |
|  |  |  |  |  |  |  |
| BLAST (% of reads) -By Species | | | | | | |
| Threshold | Sensitivity (TP/(TP+FN)) | True Positives | False Negatives | Specificity (TN/(FP+TN) | True Negatives | False Positives |
| 10.0000% | 45.5% | 5 | 6 | 100.0% | 21885 | 0 |
| 1.0000% | 63.6% | 7 | 4 | 100.0% | 21885 | 0 |
| 0.1000% | 100.0% | 11 | 0 | 99.9% | 21881 | 4 |
| 0.0100% | 100.0% | 11 | 0 | 99.6% | 21804 | 81 |
| 0.0010% | 100.0% | 11 | 0 | 98.2% | 21487 | 398 |
| 0.0001% | 100.0% | 11 | 0 | 95.6% | 20926 | 959 |

**Table S1 – Species-level Sensitivity and Specificity of MegaBLAST and MetaPhlAN, in relation to Table 1.** We used the ABRF positive control sample with 11 known organisms to characterize the sensitivity and specificity of MegaBLAST and MetaPhlAn at various thresholds (left). Notably, the two species that MetaPhlAn could not detect are not actually present in its database (for either v1.7 or 2.0). Although ROC plots do not usually to penalize a tool for something it was not designed to detect, we still kept this as a false negative since our goal is to find all species present from our metagenomic samples.

| **Supplemental Table 2: Sensitivity and Specificity of Identification Methods by Genus** | | | | | | |
| --- | --- | --- | --- | --- | --- | --- |
|  |  |  |  |  |  |  |
| MetaPhlAn (Normalized Proportion Value) | | | | | | |
| Threshold | Sensitivity (TP/(TP+FN)) | True Positives | False Negatives | Specificity (TN/(FP+TN) | True Negatives | False Positives |
| 10 | 27.3% | 3 | 8 | 100.0% | 16904 | 0 |
| 1 | 81.8% | 9 | 2 | 100.0% | 16903 | 1 |
| 0.1 | 90.9% | 10 | 1 | 99.9% | 16895 | 9 |
| 0.01 | 100.0% | 11 | 0 | 99.9% | 16894 | 10 |
| 0.001 | 100.0% | 11 | 0 | 99.9% | 16884 | 20 |
| 0.0001 | 100.0% | 11 | 0 | 99.9% | 16884 | 20 |
|  |  |  |  |  |  |  |
| BLAST (% of reads) | | | | | | |
| Threshold | Sensitivity (TP/(TP+FN)) | True Positives | False Negatives | Specificity (TN/(FP+TN) | True Negatives | False Positives |
| 10.0000% | 45.5% | 5 | 6 | 100.0% | 21885 | 0 |
| 1.0000% | 72.7% | 8 | 3 | 100.0% | 21884 | 1 |
| 0.1000% | 100.0% | 11 | 0 | 99.9% | 21883 | 2 |
| 0.0100% | 100.0% | 11 | 0 | 99.8% | 21843 | 42 |
| 0.0010% | 100.0% | 11 | 0 | 99.0% | 21676 | 209 |
| 0.0001% | 100.0% | 11 | 0 | 96.3% | 21072 | 813 |

**Table S2 – Genus-level Sensitivity and Specificity of MegaBLAST and MetaPhlAN, in relation to Table S1 (Genus vs. Species).** We used the ABRF positive control sample with 11 known organisms to characterize the sensitivity and specificity of MegaBLAST and MetaPhlAn at various thresholds. Chosen thresholds are shown in red.

| **Supplemental Table 3 - Top Eukaryotic Taxa Found** | | | | |
| --- | --- | --- | --- | --- |
|  |  |  |  |  |
| **Eukaryota** | | | | |
| **No.** | **Genus** | **Species** | **Common Name** | **NCBI TaxID** |
| 307 | *Dendroctonus* | *ponderosae* | Mountain Pine Beetle | 77166 |
| 195 | *Ceratitis* | *capitata* | Mediterranean Fruit Fly | 7213 |
| 164 | *Cucumis* | *sativus* | Cucumber | 3659 |
| 84 | *Homo* | *sapiens* | Human | 9606 |
| 32 | *Cicer* | *arietinum* | Chick Pea | 3827 |
| 18 | *Candida* | *parapsilosis* | Fungus (Yeast) | 5480 |
| 12 | *Ricinus* | *communis* | Castor Oil Plant | 3988 |
| 11 | *Culex* | *quinquefasciatus* | House Mosquito | 7176 |
| 10 | *Ixodes* | *scapularis* | Deer Tick | 6945 |
| 6 | *Equus* | *caballus* | Horse | 9796 |

**Table S3 – Top Eukaryotic Taxa Found, in relation to Table 1.** We used the BLAST thresholds to count the number of stations (No.) showing different eukaryotic species. NCBI Taxonomy ID is on the right side, after the common name.

| Supplemental Table 4:  Potential Infectious Agents & Toxins | | | | |
| --- | --- | --- | --- | --- |
|  |  |  |  |  |
| **Genus and species** | **No. Reads (MegaBLAST)** | **MetaPhlAn abundance** | **No. Samples** | **Database** |
| *Bacillus* *anthracis* | 10,048 | 0.19 | 2 | CDC |
| *Clostridium perfringens* | 89,720 | 0.16 | 10 | PATRIC |
| *Clostridium tetani* | 61,718 | 0.17 | 9 | PATRIC |
| *Escherichia coli* | 3,484,839 | 5.69 | 68 | PATRIC |
| *Shigella sonnei* | 27,163 | 0.07 | 3 | PATRIC |
| *Staphylococcus aureus* | 648,471 | 1.09 | 65 | CDC |
| *Yersinia pestis* | 36,236 | 0.01 | 3 | CDC |

**Table S4. Potential Infectious Agents and Toxins, in relation to Table 1.** Species found by MetaPhlAn and BLAST that matched the CDC or PATRIC databases for infectious agents are summarized. Results are listed according to the total number of reads used by BLAST to predict their presence, the normalized MetaPhlAn abundance, and the number of stations in which these organisms were found. Note that *Y. pestis* was found with MetaPhlAn 1.7, SURPI, and BLAST.

| Supplemental Table 5: Colony-Forming Units (CFUs) as a Function of Rider Frequency | | | | | | | | | | | |
| --- | --- | --- | --- | --- | --- | --- | --- | --- | --- | --- | --- |
|  |  |  |  |  |  |  |  |  |  |  |  |
| **Station Metadata** | | | Total CFUs (No Antibiotics) | | | |  | Total CFUs (Tetracycline Treated) | | | |
|  |  |  | Medium (TSA) | | Medium (LB) | |  | Medium (TSA) | | Medium (LB) | |
| **Subway Station** | **Riders/Day** | **Sample ID** | 28^o^C | 37^o^C | 28^o^C | 37^o^C |  | 28^o^C | 37^o^C | 28^o^C | 37  ^o^C |
| 50th St./8th Ave. | 18,776 | W0940 | 1,700 | 300 | 1,600 | 400 |  | 60 | 10 | 50 | 0 |
| 53rd St./5th Ave. | 26,667 | W0942 | 1,200 | 300 | 1,000 | 1,300 |  | 170 | 20 | 130 | 20 |
| 77th St. | 36,182 | W0930 | 4,700 | 3,600 | 3,600 | 3,600 |  | 480 | 90 | 230 | 10 |
| 68th St. Hunter College | 36,269 | W0928 | 4,700 | 1,200 | 6,000 | 1,200 |  | 150 | 70 | 260 | 90 |
| Grand Central 42nd St. | 153,747 | W0934 | 900 | 500 | 600 | 300 |  | 50 | 20 | 20 | 0 |
| Grand Central 42nd St. | 153,747 | W0932 | 1,300 | 300 | 1,200 | 1,100 |  | 770 | 10 | 670 | 0 |
| Times Square 42nd St. | 189,506 | W0936 | 30,000 | 20,700 | 30,000 | 20,300 |  | 860 | 120 | 920 | 210 |
| Times Square 42nd St. | 189,506 | W0938 | 30,000 | 30,000 | 30,000 | 30,000 |  | 2300 | 3000 | 1900 | 450 |

**Table S5 – Analysis of Colony Forming Units (CFUs) for cultured subway samples, in relation to Figure 4.** We used swabs from eight samples to test the impact of culturing on two media (LB, TSA) and two temperatures on the total CFUs detected. These samples were then sequenced and compared for species detection (**Figure 4**).

| Supplemental Table 6 - Functional Content of Subway Bacterial Gene Sequences | | | | | | |
| --- | --- | --- | --- | --- | --- | --- |
|  |  |  |  |  |  |  |
|  | **OTU Rank (per sample)** | | | |  |  |
| **Operational Taxonomic Unit (OTU)** | **P00050** | **P00052** | **P00056** | **P00061** | **KEGG_Pathways** |  |
| Transporters | 1 | 1 | 1 | 1 | Environmental Information Processing; Membrane Transport; Transporters |  |
| General function prediction only | 2 | 3 | 2 | 2 | Unclassified; Poorly Characterized; General function prediction only |  |
| ABC transporters | 3 | 2 | 3 | 3 | Environmental Information Processing; Membrane Transport; ABC transporters |  |
| Photosynthesis proteins | 5 | 54 | 4 | 4 | Metabolism; Energy Metabolism; Photosynthesis proteins |  |
| DNA repair and recombination proteins | 4 | 4 | 5 | 5 | Genetic Information Processing; Replication and Repair; DNA repair and recombination proteins |  |
| Two-component system | 6 | 5 | 7 | 6 | Environmental Information Processing; Signal Transduction; Two-component system |  |
| Peptidases | 7 | 9 | 6 | 7 | Metabolism; Enzyme Families; Peptidases |  |
| Purine metabolism | 8 | 6 | 9 | 8 | Metabolism; Nucleotide Metabolism; Purine metabolism |  |
| Photosynthesis | 10 | 63 | 8 | 9 | Metabolism; Energy Metabolism; Photosynthesis |  |
| Ribosome | 9 | 7 | 11 | 10 | Genetic Information Processing; Translation; Ribosome |  |
| Porphyrin and chlorophyll metabolism | 11 | 19 | 10 | 11 | Metabolism; Metabolism of Cofactors and Vitamins; Porphyrin and chlorophyll metabolism |  |
| Chromosome | 13 | 14 | 12 | 12 | Genetic Information Processing; Replication and Repair; Chromosome |  |
| Secretion system | 12 | 10 | 13 | 13 | Environmental Information Processing; Membrane Transport; Secretion system |  |
| Oxidative phosphorylation | 14 | 15 | 14 | 14 | Metabolism; Energy Metabolism; Oxidative phosphorylation |  |
| Function unknown | 15 | 13 | 15 | 15 | Unclassified; Poorly Characterized; Function unknown |  |
| Bacterial motility proteins | 16 | 8 | 19 | 16 | Cellular Processes; Cell Motility; Bacterial motility proteins |  |
| Pyrimidine metabolism | 18 | 12 | 16 | 17 | Metabolism; Nucleotide Metabolism; Pyrimidine metabolism |  |
| Arginine and proline metabolism | 17 | 16 | 17 | 18 | Metabolism; Amino Acid Metabolism; Arginine and proline metabolism |  |
| Others | 19 | 37 | 18 | 19 | Unclassified; Metabolism; Others |  |
| Amino acid related enzymes | 20 | 17 | 20 | 20 | Metabolism; Amino Acid Metabolism; Amino acid related enzymes |  |
| Amino sugar and nucleotide sugar metabolism | 21 | 29 | 21 | 21 | Metabolism; Carbohydrate Metabolism; Amino sugar and nucleotide sugar metabolism |  |
| Glycolysis / Gluconeogenesis | 22 | 20 | 22 | 22 | Metabolism; Carbohydrate Metabolism; Glycolysis / Gluconeogenesis |  |
| Chaperones and folding catalysts | 24 | 40 | 23 | 23 | Genetic Information Processing; Folding, Sorting and Degradation; Chaperones and folding catalysts |  |
| Pyruvate metabolism | 23 | 18 | 24 | 24 | Metabolism; Carbohydrate Metabolism; Pyruvate metabolism |  |
| Ribosome Biogenesis | 25 | 22 | 25 | 25 | Genetic Information Processing; Translation; Ribosome Biogenesis |  |
| Methane metabolism | 26 | 21 | 26 | 26 | Metabolism; Energy Metabolism; Methane metabolism |  |
| Aminoacyl-tRNA biosynthesis | 28 | 25 | 28 | 27 | Genetic Information Processing; Translation; Aminoacyl-tRNA biosynthesis |  |
| Replication, recombination and repair proteins | 29 | 42 | 27 | 28 | Unclassified; Genetic Information Processing; Replication, recombination and repair proteins |  |
| Protein folding and associated processing | 30 | 50 | 29 | 29 | Unclassified; Genetic Information Processing; Protein folding and associated processing |  |
| Transcription factors | 27 | 11 | 30 | 30 | Genetic Information Processing; Transcription; Transcription factors |  |

**Table S6. Functional Dynamics of Subway Bacterial Genes, in relation to Table 1**. OTU breakdown of a subset four subway samples based on 16S rRNA sequencing and QIIME and PICRUSt analysis.

| **Supplemental Table 7 - NYC Air and Subway Microbiome** | | | | |
| --- | --- | --- | --- | --- |
|  |  |  |  |  |
| **Taxa** | **Subway (1=Y, 0=N)** | **# of PathoMap Samples** | **AVG MetaPhlAn Abundance** | **BLAST** |
| Pseudomonas | 1 | 1355 | 36.804 | 0.22142 |
| Achromobacter | 1 | 164 | 1.016 | 0.00403 |
| Sphingomonas | 1 | 5 | 0.001 | 0.00019 |
| Klebsiella | 1 | 653 | 2.357 | 0.01527 |
| Escherichia | 1 | 875 | 3.833 | 0.00113 |
| Bradyrhizobium | 0 | 0 | 0.000 | 0.00000 |
| Sphingobium | 1 | 89 | 0.033 | 0.00030 |
| Propionibacterium | 1 | 201 | 0.101 | 0.00025 |
| Phyllobacteriaceae | 0 | 0 | 0.000 | 0.00000 |
| Bordetella | 1 | 155 | 0.618 | 0.00032 |
| Sphingopyxis | 1 | 5 | 0.001 | 0.00003 |
| Acinetobacter | 1 | 1192 | 10.630 | 0.02074 |
| Cornyebacterium | 0 | 0 | 0.000 | 0.00000 |
| Novosphingobium | 1 | 89 | 0.033 | 0.00010 |
| Variovorax | 1 | 40 | 0.006 | 0.00015 |
| Serratia | 1 | 29 | 0.473 | 0.00343 |
| Legionella | 0 | 0 | 0.000 | 0.00000 |
| Staphylococcus | 1 | 425 | 0.808 | 0.00318 |
| Enhydrobacter | 1 | 201 | 1.006 | 0.00000 |
| Chryseobacterium | 1 | 187 | 0.418 | 0.00001 |
| Rhodopirellula | 0 | 0 | 0.000 | 0.00000 |
| Synechococcus | 0 | 0 | 0.000 | 0.00000 |
| Planctomyces | 0 | 0 | 0.000 | 0.00000 |
| Flavobacterium | 0 | 0 | 0.000 | 0.00000 |
| Wolbachia | 1 | 1 | 0.002 | 0.00000 |
| Pirellula | 0 | 0 | 0.000 | 0.00000 |
| Blastopirellula | 0 | 0 | 0.000 | 0.00000 |
| Coraliomargarita | 0 | 0 | 0.000 | 0.00000 |

**Table S7 – Comparison of air-based microbiome collection, in relation to Table 1.** We compared the top ranking-families from the Venter and the Mason study of the urban microbiome and found many similar families.

**Supplemental Code and Scripts**

#This document contains all the scripts and commands used to generate the data and analysis for this paper

#For any questions please email Ebrahim Afshinnekoo (eba2001@med.cornell.edu), Cem Meydan (cem2009@med.cornell.edu) or Christopher E. Mason (chm2042@med.cornell.edu).

#TAXA CHARACTERIZATION

#MetaPhlAn 2.0 (https://bitbucket.org/biobakery/metaphlan2)

#Script was submitted using SGE platform

#Default parameters were used and output was converted into BIOM format

#!/bin/bash

#$ -cwd

#$ -j n

#$ -l h_rt=96:00:00

#$ -m bea

#$ -M ebbyafshinnekoo@gmail.com

#$ -N metaphlan

#$ -pe smp 5

#$ -l os=rhel6.3

#$ -l h_vmem=1G

rsync -a /zenodotus/masonlab/pathomap_scratch/ebrahim/Database/*fastq.gz $TMPDIR

mkdir $TMPDIR/metaphlan_out

cd $TMPDIR

for file in $(find $TMPDIR/metaphlan_out *fastq.gz)

do

zcat $file | /home/darryl/anaconda/bin/python /home/ebrahim/bin/metaphlan2/metaphlan2.py --bowtie2db /home/ebrahim/bin/metaphlan2/db_v20/mpa_v20_m200 --bowtie2_exe /home/darryl/bin/bowtie2-2.1.0/bowtie2 --input_type fastq --mpa_pkl /home/ebrahim/bin/metaphlan2/db_v20/mpa_v20_m200.pkl --nproc $NSLOTS --biom biom_output --bowtie2out $file.bt2.out > $TMPDIR/metaphlan_out/$file.biom

done

rsync -a $TMPDIR/metaphlan_out /zenodotus/masonlab/pathomap_scratch/ebrahim/

#Note if you have multiple FASTQ files per sample (paired-end and multiple lanes) then these files should be concatenated into one FASTQ per pair (SampleName_R1.fastq.gz & SampleName_R2.fastq.gz) or you can input multiple FASTQ files separating by commas.

#To combine all the output files execute the "combiner.pl" script; perl combiner.pl > results.txt

#/usr/bin/perl

@allfiles = <*>;

foreach $file (@allfiles){

($name, $part, $p1, $p2, $p3) = split ("_", $file);

if($file =~ m/PATHOMAP/i){

chomp($name);

$allnames{$name}=1;

open(FILEHERE, "<$file") or die "Could not open file called $file";

while(<FILEHERE>){

($species, $perc) = split ("\t", $_);

$$name{$species}+=$perc;

$allspecies{$species}=1;

}

}

}

@allspecies = %allspecies;

@allnames = %allnames;

@allnames = sort (@allnames);

#print header

print "Species\t";

foreach (@allnames){

if ($_ =~ m/PATHOMAP/i){

print "$_\t";

}

}

print "\n";

#print data

foreach $member (@allspecies){

print "$member\t";

foreach $sample (@allnames){

if ($sample =~ m/PATHOMAP/i){

# print "match of $sample to pathomap\n";

print "$$sample{$member}\t";

}

}

print "\n";

}

#To generate a complete master sheet of all the taxa use the compileMetaforMaster.py script with the output of the combiner.pl script (results.txt) as the input; python compileMetaforMaster.py results.txt

list_of_info = []

bacteria_info = read_bacteria(bacteria_file)

list_bacteria = len(bacteria_info)

for x in range(0,list_bacteria):

b = string.split(bacteria_info[x][0],",")

list_of_info.append(b)

return list_of_info

def compileList(biglist, smalllist):

for x in range(0,len(biglist)):

for y in range(0, len(smalllist)):

if biglist[x][0] == smalllist[y][0]:

for z in range(1,len(smalllist[y])):

biglist[x].append(smalllist[y][z])

return biglist

def bacteriaToSample(file):

samples_list = createBacteriaList(file)

print samples_list[0][0]

bacteria_sample = [] #will be list of lists

for x in range(1 , len(samples_list)):

bacteria_amount = []

bacteria_amount.append(samples_list[x][0])

for y in range(1, len(samples_list[x])):

if samples_list[x][y] != "":

bacteria_amount.append(samples_list[0][y])

bacteria_amount.append(samples_list[x][y])

bacteria_sample.append(bacteria_amount)

return bacteria_sample

def writeCSVtoFile(file, list):

with open(file, "wb") as f:

writer = csv.writer(f)

writer.writerows(list)

compiled_Metaphlan = read_bacteria(sys.argv[1])

#removes the rows that just have 1 in them

for sample in compiled_Metaphlan:

if sample[0] == "1":

compiled_Metaphlan.remove(sample)

writeCSVtoFile("bacteria and samples.csv", compiled_Metaphlan)

list_sample_amount = bacteriaToSample("bacteria and samples.csv")

writeCSVtoFile("sample and amounts.csv", list_sample_amount)

#TARGETED ALIGNMENTS

#bwa-0.7.10 (http://bio-bwa.sourceforge.net)

#samtools-0.1.19 (http://samtools.sourceforge.net)

/zenodotus/masonlab/pathomap_scratch/ebrahim/bwa-0.7.10/bwa mem -t 20 <ref_sequence.fasta> <(cat *R1*.fastq.gz) <(cat *R2*.fastq.gz) > filename.sam

/zenodotus/masonlab/pathomap_scratch/ebrahim/samtools-0.1.19/samtools view -bS -F 4 filename.sam > filename.bam

/zenodotus/masonlab/pathomap_scratch/ebrahim/samtools-0.1.19/samtools sort filename.bam filename.sorted

/zenodotus/masonlab/pathomap_scratch/ebrahim/samtools-0.1.19/samtools index filename.sorted.bam

#CALLING VARIANTS

#samtools-0.1.19 (http://samtools.sourceforge.net)

/zenodotus/masonlab/pathomap_scratch/ebrahim/samtools-0.1.19/samtools mpileup -DgSu -f <ref_sequence.fasta> filename.bam > filename.bcf

/zenodotus/masonlab/pathomap_scratch/ebrahim/samtools-0.1.19/bcftools/bcftools view -c -g -e -v filename.bcf > filename.vcf

#MAPPING COVERAGE

#bedtools-2.22.0 (http://bedtools.readthedocs.org/en/latest/)

/zenodotus/masonlab/pathomap_scratch/ebrahim/bedtools-2.22.0/bin/genomeCoverageBed -g <ref_sequence.fa> -ibam filename.sorted.bam -d > filename_cov.txt

#To combine all the coverage .txt files use this script "coverage_combiner.pl"

$allnames{$name}=1;

open(FILEHERE, "<$file") or die "Could not open file called $file";

while(<FILEHERE>){

($chromosome, $position, $cov) = split ("\t", $_);

$$name{$chromosome}+=$cov;

# $sizes{$chromosome}++;

$allchromos{$chromosome}=1;

}

}

}

@allchromos = %allchromos;

@allnames = %allnames;

@allnames = sort (@allnames);

@allsizes = %sizes;

#print "$allsizes[0], $allsizes[1]\n";

#foreach $dude (@allnames){print "$dude\n";}

#print header

#print "Chromosome/Contig\t";

foreach (@allnames){

if ($_ =~ m/P/i){

print "$_\t";

}

}

print "\n";

#print data

foreach $member (@allchromos){

print "$member\t";

# print "$member and $sizes{$member}\n";

foreach $sample (@allnames){

if ($sample =~ m/P/i && $sample != 1 && $$sample{$member}>0){

# print "match of $sample to $sample_match\n";

print "$$sample{$member}\t";

}

# elsif($sample > 1){}

# else{

# print "0\t";

# }

}

print "\n";

}

**Human Ancestry Dataset Preparation**

Each PathoMap vcf was merged with another vcf containing all the dbsnp annotations from the hg19 reference (00-All.vcf), using the vcf-merge programme from vcftools. This ensured that each dbsnp site in the PathoMap vcf was annotated.

vcf-merge 00-All.vcf.gz x.vcf.gz | bgzip -c > xmerge.vcf.gz

To remove the unwanted non-genotyped dbsnp sites in the new merged file, an isec command was performed. isec finds common elements (intersections) between vcf files, be they genomic sites or individuals. For genomic data, it operates by nucleotide locus and thus pulls the sites from the original PathoMap out, including those not present in the dbsnp database at the time.

vcf-isec -n +2 xmerge.vcf.gz x.vcf.gz | bgzip -c > xisec.vcf.gz

In order to create a 1000 Genomes compliment for the Pathomap sample, the dbsnp annotated sites from xisec.vcf.gz were obtained from its .map following conversion to a PED file.

vcftools --gzvcf xfinal.vcf.gz --plink --out x

The SNPs were extracted form the phase 2 whole genome using vcftools. The whole genome data is available to the public divided by chromosome.

vcftools --gzvcf ALL.chr1.phase1_release_v3.20101123.snps_indels_svs.genotypes.vcf.gz --snps xsnpslist --plink-tped --out xlistch1

A tped output was necessary given the scale of data involved.

Subsequently, the tpeds were merged using Plink and this merged file converted to ped format with any SNPs with a missing rate above 1% excluded.

Plink --tfile xlistch1 --merge-list 1kGenomes2-22 --geno 0.01--recode --out 1kGenomesAllx

**Admixture Analysis**

Admixture analysis was performed for each PathoMap sample.

Each PathoMap ped, x was merged with it's complimentary 1kGenomesAllx and the merged file output as a 12 ped.

Plink --file x merge 1kGenomesAllx.ped 1kGenomesAllx.map --recode12 –out x+1kGenomesAllx12

plink --file x+1kGenomesAllx12 --make-bed --out x+1kGenomesAllx12

The resulting ped file was subsequently run by admixture with a k value of 4 to correspond roughly to a genetic division based on Sub-Saharan African, European, East Asian and Amerindian, as these were the general population divisions in the populations present in the 1000 Genomes. However, the Amerindian component was only represented by a highly European admixed Mexican sample. Despite this, an identifiable Amerindian component was observed.

admixture x+1kGenomesAllx12.bed 4

The subsequent x+1kGenomeAllx12.4.Q was plotted in R as a box plot.
